# Experimental Infection with *Mycoplasma hyopneumoniae* Strain 232 in Swine Influences the Lower Respiratory Microbiota

**DOI:** 10.3390/vetsci9120674

**Published:** 2022-12-05

**Authors:** Henrique Meiroz de Souza Almeida, Karina Sonalio, Marina Lopes Mechler-Dreibi, Fernando Antônio Moreira Petri, Gabriel Yuri Storino, Dominiek Maes, Luís Guilherme de Oliveira

**Affiliations:** 1 School of Agricultural and Veterinarian Sciences, São Paulo State University (Unesp), Jaboticabal 14884-900, São Paul, Brazil; 2 Unit of Porcine Health Management, Faculty of Veterinary Medicine, Ghent University, Salisburylaan 133, 9820 Merelbeke, Belgium

**Keywords:** enzootic pneumoniae, alpha diversity, beta diversity, respiratory tract, pigs

## Abstract

**Simple Summary:**

*Mycoplasma hyopneumoniae* is a bacterium that causes pneumonia in pigs and can facilitate the establishment of other respiratory diseases. The microbiota, which comprises the microorganisms found in a specific environment (in this case, the respiratory tract), can be altered by the presence of several pathogens. Therefore, knowing how *M. hyopneumoniae* can affect the microbiota of the lower respiratory tract and nasal turbinates in experimentally infected pigs is important to understand the interaction between microorganisms that could lead to the development of disease. Thus, we investigated the bacterial composition of the lungs and noses of infected and non-infected pigs. The results showed that the lungs of infected pigs were mostly colonized (growth and multiplication of a microorganism) by *M. hyopneumoniae*, and there were not many other species in the lungs. In contrast, in the non-infected pigs, a more diverse lower respiratory microbiota was observed, meaning that there were more species of bacteria in the non-infected pigs than in the infected ones. No differences were observed between the nose microbiota in infected and non-infected pigs. In conclusion, this pathogen can alter the number of bacterial species in the lungs, which could result in more respiratory problems in swine production.

**Abstract:**

*Mycoplasma (M.) hyopneumoniae*, the etiological agent of swine enzootic pneumonia, has been reported to increase the susceptibility to secondary infections and modulate the respiratory microbiota in infected pigs. However, no studies have assessed the influence of *M. hyopneumoniae* on the respiratory microbiota diversity under experimental conditions. Therefore, this study evaluated the impact of *M. hyopneumoniae* infection on the respiratory microbiota of experimentally infected swine over time. To accomplish this, 12 weaned pigs from a *M*. *hyopneumoniae*-free farm were divided into two groups: *M. hyopneumoniae* strain 232 infected (n = 8) and non-infected (n = 4). The first group received 10 mL of Friis medium containing 10^7^ CCU/mL of *M. hyopneumoniae* while the control group received 10 mL of sterile Friis medium. Inoculation of both groups was performed intratracheally when the animals were 35 days old (d0). At 28 days post-inoculation (dpi) and 56 dpi, 4 infected animals plus 2 controls were humanely euthanized, and biopsy samples of nasal turbinates (NT) and bronchus-alveolar lavage fluid (BALF) samples were collected. The DNA was extracted from the individual samples, and each group had the samples pooled and submitted to next-generation sequencing. Taxonomic analysis, alpha and beta diversity indexes, weighted unifrac, and unweighted unifrac distances were calculated. A high relative frequency (99%) of *M. hyopneumoniae* in BALF samples from infected animals was observed with no significant variation between time points. The infection did not seem to alter the diversity and evenness of bacterial communities in NT, thus, *M. hyopneumoniae* relative frequency was low in NT pools from infected animals (28 dpi—0.83%; 56 dpi—0.89%). PCoA diagrams showed that BALF samples from infected pigs were grouped and far from the control samples, whereas NT from infected animals were not separated from the control. Under the present coditions, *M. hyopneumoniae* infection influenced the lower respiratory microbiota, which could contribute to the increased susceptibility of infected animals to respiratory infections.

## 1. Introduction

*Mycoplasma (M.) hyopneumoniae* is a respiratory pathogen of swine, with a worldwide distribution. Infections cause chronic pneumonia, dry, non-productive cough, and a reduction in average daily weight gain [1]. The agent is known for colonizing the ciliated epithelial lining of the respiratory tract of swine with consequent cilia disruption leading to an increased susceptibility to secondary infections, as well as synergistic action with bacterial and viral pathogens in the porcine respiratory disease complex (PRDC) [2]. The interaction between respiratory pathogens plays an important role in the PRDC, as changes in the microbiome diversity can result in dysbiosis and disease development.

The microbiome is the community of microorganisms that live on the mucosal surface and/or the skin of animals [3]. The presence of such microorganisms’ communities in the organs and tissues of mammals was shown to enhance immune system stimulation, resulting in stronger local mucosal immunity [4]. The greater diversity of the commensal microbiota in lung airways seems to be an important factor involved in the development of antiviral immunity against influenza in mice [5]. However, the influence of other pathogens like *M. hyopneumoniae* in the composition of lung and nasal microbiota under experimental conditions remains to be investigated, and additional studies are required for a better understanding [3].

Infections caused by respiratory pathogens were shown to greatly affect the diversity and composition of the oropharyngeal microbiota in pigs, where the results indicate that *Streptococcus, Lactobacillus* and *Actinobacillus* are the main components of the microbiome in healthy pigs, whereas *Moraxella, Veillonella* and *Porphyromonas* are suggested to play a potential role in porcine respiratory diseases [6]. As reported by Slifierz [7], the diversity and richness of the nasal microbiota in healthy animals seem to stabilize 2–3 weeks after weaning. Regarding the lower respiratory tract, previously published studies detected changes in the diversity and microbiota composition of swine lungs, where gross lesions were suggestive of *M. hyopneumoniae* infection under field conditions [8,9]. However, since little is known about the impact of *M. hyopneumoniae* infection in the lower respiratory tract and nasal microbiota of swine under experimental conditions, this study focused on assessing the abovementioned conditions in experimentally infected swine.

## 2. Materials and Methods

### 2.1. Experimental Design and Animal Inoculation

A group of 12 male Large White pigs, weaned at 28 days of age and originating from a certified *M. hyopneumoniae*-free farm, were randomly allocated into two different groups, four in the non-infected, or control group (CG), and eight in the infected group (IG). Both groups were kept in separate, but similar facilities, with feed and water *ad libitum*, and were able to acclimatize for a week. During the acclimation period, at dpi −7, serum (ELISA) and laryngeal swab samples (qPCR) were collected from all animals and tested, confirming their *M. hyopneumoniae*-free status.

At the day of inoculation, when the piglets were 35 days of age (i.e., day 0 post-inoculation (dpi)), the eight animals in the IG group were inoculated with 10 mL of Friis medium containing 10^7^ CCU/mL of *M. hyopneumoniae*, strain 232 [10], using an intratracheal catheter (Embraco N°5, Joinville, Santa Catarina, Brazil), as described elsewhere [11,12,13]. Briefly, the catheter was inserted approximately 18 cm in to the trachea. The inoculum was administered followed by 10 mL of sterile saline solution. The same procedure was performed with the 4 animals in the CG group, but using 10 mL of sterile Friis broth, followed by the administration of 10 mL of sterile saline solution.

This study was approved by the Ethical Committee in Animal Use of the School of Agricultural and Veterinary Sciences, São Paulo State University, Campus Jaboticabal, under protocol # 9.952/16.

### 2.2. Necropsy, Sample Collection, and Lung Lesion Scoring

At 28 dpi and 56 dpi, 2 CG and 4 IG animals were humanely euthanized according to the guidelines of the Brazilian Federal Veterinary Medicine council. Immediately after death, the noses of the animals were transversally sawed using a disinfected saw and both nasal turbinates (NT) were exposed. Then, 1 cm of tissue from each turbinate was collected with the aid of sterilized forceps and scalpel blades. Samples were stored in sterile DNase and RNase-free cryovials (Corning, New York, NY, USA), flash-frozen in liquid nitrogen and stored at −80 °C until processing.

After NT collection, the trachea and lungs were removed for bronchus alveolar lavage fluid (BALF) collection and macroscopic lung lesion evaluation. For BALF sampling, an incision was made in the trachea, approximately 5 cm before the trachea bifurcation, and with the aid of a sterilized plastic pipette and automatic pipettor, 20 mL of sterilized PBS (1X, pH 7.4; Sigma-Aldrich, Darmstadt, Germany) were dispensed into the bronchus. The lung was slightly massaged for 10 s, and the liquid was aspirated back using the pipette. Then, BALF samples were stored in duplicates in 2.0 mL DNase and RNase-free cryovials (Corning, New York, NY, USA), flash-frozen in liquid nitrogen and kept at −80 °C until they were used. Macroscopic *M. hyopneumoniae*-like lung lesions were evaluated, as described by Straw et al. [14]. The score varied from 0% to 100%, or from no lesions to entire lung affected, respectively.

Afterward, 2 cm^2^ lung samples comprising both affected and non-affected tissue were collected and stored in 10% buffered formalin for histopathological evaluation and scoring, as described by Livingston et al. [15]. Lastly, the weight of the animals was assessed every four weeks (0, 28 and 56 dpi) to calculate the average daily weight gain (ADWG). To do so, the initial weight was subtracted from the final weight, and the product was divided by the number of days between 0 dpi and the final weight (28 or 56 dpi).

### 2.3. DNA Extraction and Quantification of Mycoplasma sp. in BALF and NT Samples

To evaluate *M. hyopneumoniae*, *M. hyorhinis* and *M. flocculare* bacterial loads in the individual BALF and NT samples, total DNA was extracted following an *in-house* protocol, as described by Kuramae-Izioka et al. [16], with modifications [12]. Purity and concentration were assessed with the aid of the Nanodrop^®^ 2000 spectrophotometer (Thermo Fisher, Waltham, MA, USA). DNA samples were stored at −20 °C until they were used. The qPCR assay was performed as previously described by Fourour et al. [17] and Ferreira et al. [18], following the MIQE guidelines [19].

### 2.4. Metagenomic DNA Extraction from NT and BALF Samples

BALF samples were thawed and centrifuged at 4 °C (Centrifuge 5804 R, Eppendorf, Hamburg, Germany) at 12,000× *g* for 20 min. The supernatant was discarded, and the pellet that was formed was used for DNA extraction. Total DNA was extracted from 0.05 g of the NT and the BALF pellet using the DNeasy Blood and Tissue (Qiagen, Germantown, MD, USA) commercial extraction kit, according to the manufacturer’s instructions. The purity and quantification of the extracts were assessed using a Nanodrop^®^ 2000c spectrophotometer (Thermo Fisher, Waltham, MA, USA).

The samples from control and infected pigs of each dpi (28 and 56) were pooled in equimolar amounts according to the concentration (ng/µL) obtained from spectrophotometry. The control pools were named: non-infected nasal turbinate 28 dpi (NINT28); non-infected nasal turbinate 56 dpi (NINT56); non-infected BALF 28 dpi (NIBALF28); and non-infected BALF 56 dpi (NIBALF56). The pools from infected animals were identified as infected nasal turbinate 28 dpi (INT28); infected nasal turbinate 56 dpi (INT56); infected BALF 28 dpi (IBALF28); and infected BALF 56 dpi (IBALF56). Finally, the integrity and quality of the pooled DNA samples were assessed using a Byoanalizer^®^ (Agilent Technologies, California, USA) before being submitted to Illumina sequencing.

### 2.5. Pooled Samples Library Preparation, Normalization, Pooling and Sequencing

Sample libraries were prepared using reagents and procedures according to the manufacturer’s instructions (Illumina, 2013). Previously published primers targeting the hypervariable region V3–V4 of the 16s bacterial rDNA [20], which results in a 465 bp amplicon, were used.

The library validation was performed in a Bioanalyzer 2100 (Agilent Technologies, California, USA), and quantified using the Kappa Library Quant Kit for Illumina (Illumina Inc., San Diego, CA, USA), according to the manufacturer’s instructions [21]. Sequencing was performed using MiSeq Reagent Kit v3 (600 cycles) (Illumina Inc., San Diego, CA, USA) in an Illumina MiSeq platform (Illumina Inc., San Diego, CA, USA), following a previously published protocol [22].

### 2.6. Data Analysis

The sequencing data that were generated were demultiplexed using the Illumina bcl2fastq (v2.19.1.403) software, and the sequencing quality assessment was performed using the DADA2 software [23]. Briefly, sequence reads were aligned and assembled in silico using the pipeline Quantitative Insights Into Microbial Ecology-QIIME2 (Version 2019.10) [24]. To estimate species diversity within samples, the sequences were rarefied at 11,150 depth. The following metrics were assessed for alpha-diversity analysis: operational taxonomic unit quantities (OTUs), Faith, Shannon, Pielou and Simpson indexes. While for beta-diversity analysis, a phylogenetic tree was generated using the Mafft (multiple sequences alignment) and FastTree software, executed under default settings.

Principal coordinates analysis of weighted unifrac and unweighted unifrac were performed to detect similarities between treatment (infected and non-infected) and time (28 dpi and 56 dpi), and based on the metrics, plots were generated using QIIME2 (Version 2019.10). Only the taxa present at >0.1% of all 16S rRNA sequences in each pool were considered. Taxonomic analysis was performed using the SILVA (release 132) database. Sequences were classified using the Naive Bayes classification of reference sequences, which were clustered with 99% similarity and trimmed to include only the V3–V4 region, limited by the primer pair used in the sequencing. For features classification, QIIME2 software was used.

## 3. Results

### 3.1. Clinical and Zootechnical Parameters

No macroscopic lung lesions were observed in the control group, while the average in the IG at 28 dpi was 15.8, reducing to 6.3 at 56 dpi. Regarding the microscopic evaluation, higher score values were seen in the IG when compared to the CG. The ADWG values in the IG were similar at both time points, whereas a numerically higher value was observed in the CG at 56 dpi. Detailed results are shown in Table 1.

### 3.2. M. hyopneumoniae, M. hyorhinis, and M. flocculare DNA Quantification in BALF and NT Samples

*M. hyopneumoniae* was not detected in BALF or NT samples from individual animals in the CG, nor in the NT samples from the IG. Not surprisingly, 1.3 × 10^6^ and 4.9 × 10^5^ copies/µL of *M. hyopneumoniae* DNA were detected in the BALF samples from the IG at 28 and 56 dpi, respectively. *M. hyorhinis* was detected in the BALF samples of both groups (IG and CG) only at 28 dpi, whereas for NT samples, the pathogen was detected at both time points but only in the IG. Lastly, *M. flocculare* was detected only in the NT samples at 56 dpi. Average quantification values are presented in Table 2.

### 3.3. Next-Generation Sequencing (NGS) Quality Assessment and Alpha Diversity Indexes from Pooled Samples

Results from NGS identified a total of 3621 features (operational taxonomic unit quantities-OTUs) with a sequence size of 465 bp. One pool (NINT28) presented a lower reads number (31,312) when compared to others. The OTUs and reads obtained for each pool are described in Table 3.

Although the samples IBALF28 and IBALF56 presented a high number of reads, there was a low number of OTUs after rarefication (43 OTUs and 51 OTUs, respectively), indicating a low species diversity in both samples. The nasal turbinate pools (NINT28, NINT56, INT28, and INT56) presented an acceptable OTUs quantity and variation, except for sample NINT28, which had a low OTUs count (29), likely due to the low yield of the sequencing (31,312 reads).

Regarding the alpha diversity metrics calculated for each pool, the Faith, Shannon, Pielou and Simpson indexes for IBALF28 and IBALF56 presented low values, influenced by the low number of OTUs. The values obtained in the calculation of each Alpha diversity metrics are also shown in Table 3.

### 3.4. Principal Coordinates Analysis (PCoA) of BALF and NT Samples

**Figure 1:**
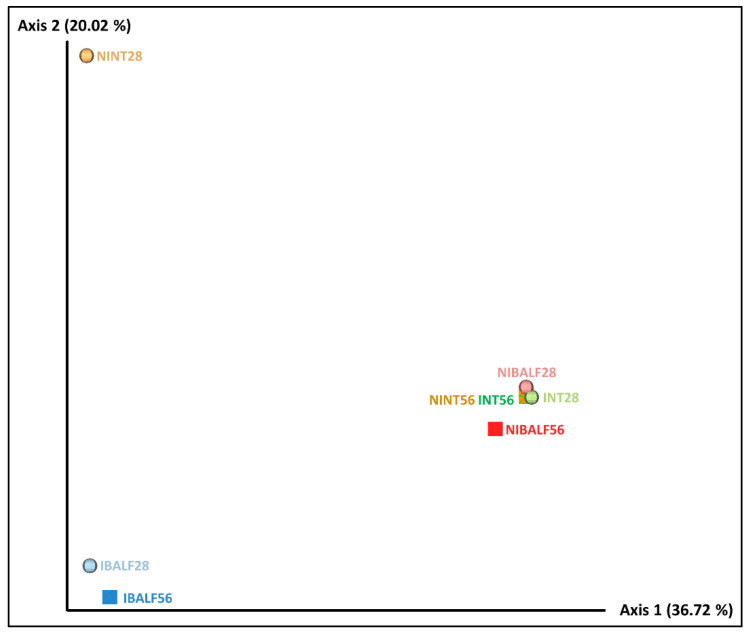
Unweighted unifrac distance PCoA diagrams with pooled nasal turbinate (NT) and bronchus-alveolar lavage fluid (BALF) samples of infected and non-infected animals at 28 and 56 days post-inoculation (dpi). NINT28 = non-infected NT 28 dpi; NINT56 = non-infected NT 56 dpi; NIBALF28 = non-infected BALF 28 dpi; NIBALF56 = non-infected BALF 56 dpi; INT28 = infected NT 28 dpi; INT56 = infected NT 56 dpi; IBALF28 = infected BALF 28 dpi; IBALF56 = infected BALF 56 dpi.

The unweighted unifrac distance-based PCoA resulted in two main separate clusters. The first one was composed of IBALF28 and IBALF56, while the other one was composed of NIBALF 28 dpi, NIBALF 56 dpi, NINT56, INT28 and INT56 samples, indicating a qualitative similarity between bacterial types in the grouped samples. NINT28 was isolated from the abovementioned clusters, however, considering axis 1, which explained the majority of the variation, it was close to IBALF28 and IBALF56, as observed in Figure 1.

**Figure 2:**
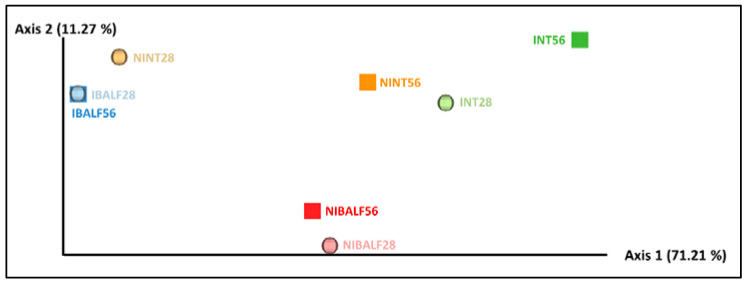
Weighted unifrac distance diagrams with pooled nasal turbinate (NT) and pooled bronchus-alveolar lavage fluid (BALF) samples of infected and non-infected animals at 28 and 56 days post-inoculation (dpi). NINT28 = non-infected NT 28 dpi; NINT56 = non-infected NT 56 dpi; NIBALF28 = non-infected BALF 28 dpi; NIBALF56 = non-infected BALF 56 dpi; INT28 = infected NT 28 dpi; INT56 = infected NT 56 dpi; IBALF28 = infected BALF 28 dpi; IBALF56 = infected BALF 56 dpi.

The weighted unifrac distance-based PCoA presented the axis with the highest value (71.21%), which explained the majority of the variance compared to the others. Two main clusters were noted, the first being composed of IBALF28 and IBALF56, and the second composed of NIBALF 56 dpi and NIBALF 28 dpi, indicating a quantitative similarity in the bacterial communities between samples. The other samples were dispersed along axis 1 of the diagram, as shown in Figure 2.

### 3.5. Taxonomic Analysis

**Figure 3:**
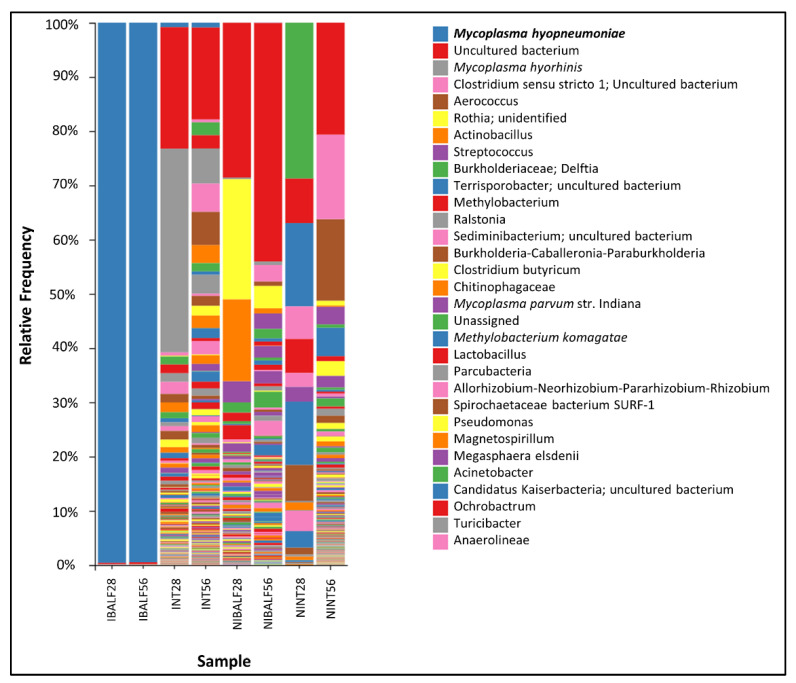
Taxonomic analysis with relative frequencies of each bacterial genera from pooled nasal turbinate and BALF samples of infected and control animals at 28 and 56 dpi.

The taxonomic analysis showed a significant predominance of *Mycoplasma hyopneumoniae* in IBALF28 e IBALF56, with a relative frequency of 99% in both time point samples (99.60 and 99.50%, respectively). The nasal turbinate pools INT28 and INT56 presented relative frequencies of *M. hyopneumoniae* of 0.83% and 0.90%, respectively. There was no detection of *M. hyopneumoniae* in NINT28, NINT56, NIBALF 28 dpi and NI BALF 56 dpi. *M. hyorhinis* was reported in INT28, NIBALF28 and NIBALF56, with a relative frequency of 37.56%, 0.29% and 0.67%, respectively. *M. flocculare* had a frequency of 0.79% in NIBALF28 and 0.10% in NIBALF56. The main taxonomic results of each pool, with genera and species (only for *Mycoplasmas* spp.), are shown in Figure 3.

*Lactobacillus* sp. was not detected in BALF samples of the IG, while it was detected at a relative frequency of 2.68% and 2.21% in NIBALF28 and NIBALF56, respectively. Similarly, the relative frequencies of *Actinobacillus* and *Streptococcus* in NIBALF28 and NIBALF56 was 15.80% and 1.18%, and 3.91% and 3.26%, respectively.

At the phylum level, differences in the relative abundance were observed between infected and non-infected samples, despite sample type. In the CG, the most abundant phylum were Proteobacteria, Firmicutes and Actinobacteria. In contrast, in the IG the phylum Tenericutes was by far the most abundant (>90% in BALF), followed by Proteobacteria (41.86% in NT) and Bacteroidetes (12.71% in NT) (Figure S1).

## 4. Discussion

In this study, *M. hyopneumoniae* experimental infection considerably reduced the number of OTUs detected in BALF at both time points, when compared to control animals. This result is reinforced by the low Faith indexes found in both inoculated group pools (28 dpi—22.8; 56 dpi—30.15), compared to the control pools (28 dpi—302.83; 56 dpi—314.36), showing a sharp reduction of microbial diversity in experimentally inoculated animals. 

The taxonomic analysis of experimentally infected animal BALF pools at both time points (28 dpi and 56 dpi) resulted in a high relative frequency of *M. hyopneumoniae*, which was also confirmed by the closeness to 0 values found by Pielou’s and Simpson’s indexes. Furthermore, it is likely that the high relative frequency observed in the BALF samples from infected animals was mostly due to the experimental infection model and the high virulence of the challenge strain (232). Similarly, a previous study using shotgun genome sequencing showed similar results of over 90% relative frequency of *M. hyopneumoniae* in pooled samples of affected lungs from field animals [8]. This fact was also reported by previous studies that detected a particular predominance of *M. hyopneumoniae* over other bacteria in swine lungs with swine enzootic pneumonia (SEP)-like lesions; these studies assessed the microbiota of naturally infected pigs during the finishing phase and at slaughter [9,25,26]. In contrast, healthy lung samples from pigs raised in commercial farms were shown to have a more diverse bacterial population when compared to lung samples of *M. hyopneumoniae*-infected pigs [8], reinforcing the evidence that *M. hyopneumoniae* infection has a negative impact on lung health due to losses in microbiota diversity. In our study, the genera *Lactobacillus*, *Streptococcus and Actinobacillus*, which have been associated with a healthy oropharyngeal microbiota [6], were not detected in BALF samples in the IG, suggesting that *M. hyopneumoniae* infection may have played a role in the colonization of the abovementioned genera in the infected lungs.

Due to its small genome and low metabolic capacity, *M. hyopneumoniae* is a fastidious growing bacterium and a poor competitor for nutrients and adhesion sites in comparison with other bacteria [10,27]. Furthermore, immune modulation abilities previously reported are responsible for producing a prolonged chronic inflammation, enhancing infection success rates [28,29]. In a related study, we reported that the expression of pro-inflammatory cytokines (IL-1, IL-1, IL-6, IL-8 and TNF-α) and the suppression of the anti-inflammatory cytokine (IL-10) in lung lesions were probably responsible for the macroscopic and microscopic lung lesions, reflecting the health status of the lung [12]. Moreover, inflammation is one of the factors that could potentially impact the local microbiota [30], playing an important role in reducing competition for adhesion sites and nutrients by eliminating competitors present in the healthy lung microbiota, which creates a better environment for *M. hyopneumoniae* to establish and grow. Because this study is part of a greater project and focused only on the respiratory microbiota diversity under experimental conditions, complementary and detailed data on the immune responses upon challenge are described elsewhere [12]. 

In contrast with BALF results, *M. hyopneumoniae* infection showed little influence on nasal microbiota diversity in this study, as demonstrated by the alpha diversity indexes, the OTUs number of NT pools and DNA quantification by qPCR. This could be related to the low relative frequency of the agent detected in such pools with little variation over time, as shown by the taxonomic analysis. However, it is fairly documented in the scientific literature that *M. hyopneumoniae* shows a tropism for lower parts of the respiratory tract of swine [31], being present in quantities 100 times more significant in lower parts of the respiratory tract compared to the nasal cavities [32]. Furthermore, the route of infection may have contributed to the lower detection of *M. hyopneumoniae* in NT samples as challenge infection happened in the trachea. Consequently, it is unlikely that *M. hyopneumoniae* would exert a significant impact on the microbiota of the upper respiratory tract in this study. 

The PCoA diagram based on the unweighted unifrac distance showed that there were no critical differences among NT pools, of either infected or not, and the non-infected BALF pools. However, despite being separated from other samples, infected BALF pools from 28 dpi and 56 dpi were close to each other along the main axis, indicating a high similarity of bacterial composition between samples, therefore confirming that for BALF samples, infection status was the main variable involved in the dissimilarity of bacterial communities. The predominance of *M. hyopneumoniae* in both BALF-infected pools severely affected the diversity and abundance of the microorganisms in the community. However, this was not observed in nasal turbinate pools, as shown in the PCoA using weighted unifrac distances. A similar abundance of microorganisms was noted in non-infected animals’ pools and in infected nasal turbinate pools, indicating that in the latter, the presence of *M. hyopneumoniae* did not impact microorganism abundance.

It has been documented that the peak of lesions and clinical signs after *M. hyopneumoniae* infection is 28 dpi [1,33]. However, the time frame (from 28 dpi to 56 dpi) did not influence microbial diversity and abundance. Alpha indexes and OTUs quantities had little variation in control and inoculated animals, which were similar in terms of time. Considering that a previous study tracking nasal microbiota changes in the early life of swine showed that after 2–3 weeks post-weaning, the microbiota of healthy animals seemed to reach a developmental milestone, it is possible that the microbiota were already established when sampling was performed, explaining the low variation found in the microbiota between sampled time points.

Regarding the multiplex qPCR analysis, the results indicate no presence of *M. hyopneumoniae* DNA in NT samples of the IG, contradicting the results of the taxonomic analysis, where *M. hyopneumoniae* was detected at a frequency of 0.8% and 0.9%. However, it is known that any PCR test may produce false-negative results if the pathogen load is below the detection limit [19] and that high-throughput sequencing has greater efficiency and can be used without prior knowledge of the specific target region, unlike PCR [22,34]. For the two other targets, *M. hyorhinis* was detected in BALF of both groups at 28 dpi and in NT samples of the infected group at both time points, while *M. flocculare* was detected only in NT samples at 56 dpi. These results also diverge from the relative frequency observed in the taxonomic analysis, which could be due to the different DNA extraction methods used for the different analyses. Furthermore, other limitations of the applied molecular techniques, such as the initial abundance of DNA and the presence of PCR inhibitors, could have played a role [16,19,34]. 

The present research showed the influence of *M. hyopneumoniae* 232 experimental infections in the respiratory tract microbiota, and consequently, the extrapolation of the results to animals raised under field conditions should be done with caution. Additionally, the results of non-infected nasal turbinate at 28 dpi were significantly affected by the low number of reads in the sequencing, which could have influenced the outcome. Considering that the *M. hyopneumoniae* 232 strain used was previously characterized as moderate virulence [10], the results could potentially change according to the strain virulence. 

In our study, pooling samples were also necessary to limit the cost of the analysis. In addition, the results regarding the microbiome analysis should be interpreted with caution as we evaluated the lung composition under experimental conditions, and therefore, other infection doses might lead to different disease outcomes and other bacteria genera. Lastly, background contamination in the microbiome analysis should be considered as control samples were not sequenced.

To the best of our knowledge, this is the first report of respiratory microbiota diversity in *M. hyopneumoniae* experimentally-infected pigs using next-generation sequencing. However, further research investigating the susceptibility of lung microbiota to *M. hyopneumoniae* infection would bring relevant information about disease establishment in relation to lung microbiome conditions.

## 5. Conclusions

*Mycoplasma hyopneumoniae* (232) experimental infection seemed to alter the microbial diversity and evenness in the lower respiratory tract of the pigs when compared to the non-infected animals, whereas the nasal microbiota did not seem to be affected by the infection, which may be due to the fact that *M. hyopneumoniae* shows tropism for the lower respiratory tract of pigs.

## Figures and Tables

**Table 1:** Mean values ± standard deviation (SD) of macroscopic lung consolidated lesions (MLCL), microscopic lung lesion scores and average daily weight gain (ADWG) values according to group and day post-inoculation (dpi).

Group	dpi	MLCL			Microsc. Score			Period	ADWG (g)		
		mean	±	SD	mean	±	SD		mean	±	SD
**Infected**	28	15.8	±	6.2	3.3	±	1.0	0–28	398	±	86
	56	6.3	±	4.1	4.0	±	0.0	0–56	398	±	45
**Control**	28	0.0	±	0.0	2.0	±	0.0	0–28	402	±	38
	56	0.0	±	0.0	0.0	±	0.0	0–56	472	±	38

**Table 2:** Mean *M. hyopneumoniae*, *M. hyorhinis*, and *M. flocculare* DNA quantification values (copies/µL) in bronchus-alveolar lavage fluid (BALF) and nasal turbinate (NT) samples based on treatment group and day post-inoculation (dpi).

Group	dpi	M. hyopneumoniae *		M. hyorhinis *		M. flocculare *	
		BALF	NT	BALF	NT	BALF	NT
**Infected**	28	1.3 × 10^6^	0.0 × 10^0^	3.1 × 10^1^	4.95 × 10^1^	0.0 × 10^0^	0.0 × 10^0^
	56	7.8× 10^5^	0.0 × 10^0^	0.0 × 10^0^	3.68 × 10^1^	0.0 × 10^0^	8.8 × 10^1^
**Control**	28	0.0 × 10^0^	0.0 × 10^0^	2.6 × 10^2^	0.0 × 10^0^	0.0 × 10^0^	0.0 × 10^0^
	56	0.0 × 10^0^	0.0 × 10^0^	0.0 × 10^0^	0.0 × 10^0^	0.0 × 10^0^	0.0 × 10^0^

* values are expressed in copies/µL.

**Table 3:** Next-generation sequencing (NGS) quality control data, operational taxonomic unit quantities (OTUs) and alpha diversity indexes (Faith, Shannon, Pielou, and Simpson) obtained from pooled nasal turbinate (NT) and bronchus-alveolar lavage fluid (BALF) samples of both infected and control animals at 28 and 56 days-post inoculation (dpi).

Group	Sample Type	dpi	# of Reads	OTUs	Faith	Shannon	Pielou	Simpson
**Infected**	NT	28	439,364	934	302.83	5.87	0.63	0.92
		56	147,576	730	314.36	7.21	0.77	0.98
	BALF	28	1,320,872	172	22.80	0.14	0.03	0.02
		56	1,067,710	214	30.15	0.17	0.03	0.03
**Control**	NT	28	31,312	29	2.50	3.45	0.71	0.85
		56	696,228	1299	295.20	6.50	0.68	0.98
	BALF	28	515,526	1125	347.63	5.85	0.61	0.86
		56	315,152	679	213.00	7.09	0.78	0.95

## Data Availability

The dataset supporting the conclusions of this article is included within the article as Supplementary Materials File S1 and Figure S1.

## Supplementary Materials

The following supporting information can be downloaded at: https://www.mdpi.com/article/10.3390/vetsci9120674/s1, File S1: Detailed information on the taxonomic analysis with relative frequencies of each bacterial genera from pooled nasal turbinate (NT) and bronchus-alveolar fluid (BALF) samples. Figure S1: Taxonomic analysis with relative frequencies of each phylum from pooled nasal turbinate and BALF samples of infected and control animals at 28 and 56 dpi.
