# Supplementary material for: Experimental Infection with Mycoplasma hyopneumoniae Strain 232 in Swine Influences the Lower Respiratory Microbiota
Source: Vet Sci. 2022 Dec 5;9(12):674. doi: 10.3390/vetsci9120674 (PMC9788024; doi:10.3390/vetsci9120674)
Supplement: Supplementary file 1 [file vetsci-09-00674-s001.zip › vetsci-2010943-supplementary File S1.pdf]

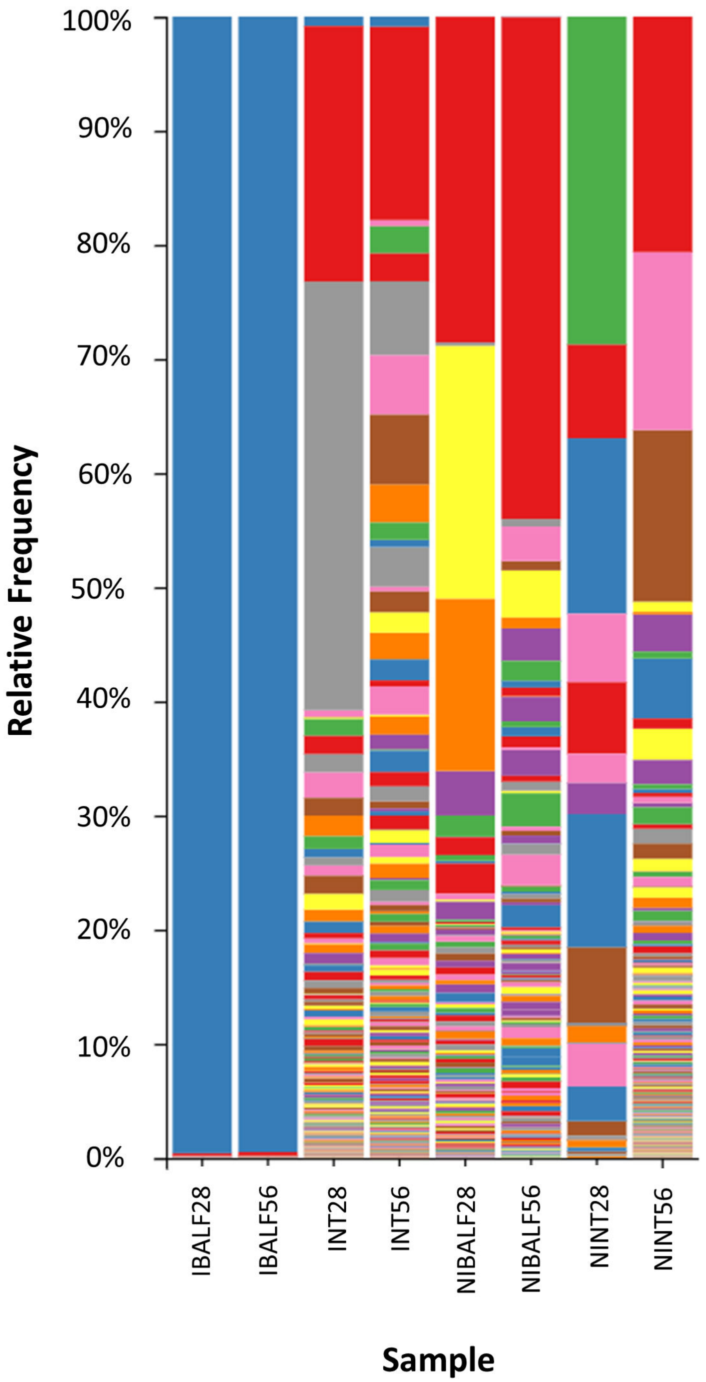
100%
90%
80%
70%
60%
50%
40%
30%
20%
10%
0%
Relative Frequency
IBALF28
IBALF56
INT28
INT56
NIBALF28
NIBALF56
NINT28
NINT56
Sample

D_0__Bacteria;D_1__Tenericutes;D_2__Mollicutes;D_3__Mycoplasmatales;D_4__Mycoplasmataceae;D_5__Mycoplasma;D_6__Mycoplasma hyopneumoniae 232

D_0__Bacteria;__;__;__;__;__;__

D_0__Bacteria;D_1__Tenericutes;D_2__Mollicutes;D_3__Mycoplasmatales;D_4__Mycoplasmataceae;D_5__Mycoplasma;D_6__Mycoplasma hyorhinis

D_0__Bacteria;D_1__Firmicutes;D_2__Clostridia;D_3__Clostridiales;D_4__Clostridiaceae 1;D_5__Clostridium sensu stricto 1;D_6__uncultured bacterium

D_0__Bacteria;D_1__Firmicutes;D_2__Bacilli;D_3__Lactobacillales;D_4__Aerococcaceae;D_5__Aerococcus;__

D_0__Bacteria;D_1__Actinobacteria;D_2__Actinobacteria;D_3__Micrococcales;D_4__Micrococcaceae;D_5__Rothia;D_6__unidentified

D_0__Bacteria;D_1__Proteobacteria;D_2__Gammaproteobacteria;D_3__Pasteurellales;D_4__Pasteurellaceae;D_5__Actinobacillus;__

D_0__Bacteria;D_1__Firmicutes;D_2__Bacilli;D_3__Lactobacillales;D_4__Streptococcaceae;D_5__Streptococcus;__

D_0__Bacteria;D_1__Proteobacteria;D_2__Gammaproteobacteria;D_3__Betaproteobacteriales;D_4__Burkholderiaceae;D_5__Delftia;__

D_0__Bacteria;D_1__Firmicutes;D_2__Clostridia;D_3__Clostridiales;D_4__Peptostreptococcaceae;D_5__Terrisporobacter;D_6__uncultured bacterium

D_0__Bacteria;D_1__Proteobacteria;D_2__Alphaproteobacteria;D_3__Rhizobiales;D_4__Beijerinckiaceae;D_5__Methylobacterium;__

D_0__Bacteria;D_1__Proteobacteria;D_2__Gammaproteobacteria;D_3__Betaproteobacteriales;D_4__Burkholderiaceae;D_5__Ralstonia;__

D_0__Bacteria;D_1__Bacteroidetes;D_2__Bacteroidia;D_3__Chitinophagales;D_4__Chitinophagaceae;D_5__Sediminibacterium;D_6__uncultured bacterium

D_0__Bacteria;D_1__Proteobacteria;D_2__Gammaproteobacteria;D_3__Betaproteobacteriales;D_4__Burkholderiaceae;D_5__Burkholderia-Caballeronia-Paraburkholderia;__

D_0__Bacteria;D_1__Firmicutes;D_2__Clostridia;D_3__Clostridiales;D_4__Clostridiaceae 1;D_5__Clostridium sensu stricto 1;D_6__Clostridium butyricum

D_0__Bacteria;D_1__Bacteroidetes;D_2__Bacteroidia;D_3__Chitinophagales;D_4__Chitinophagaceae;__;__

D_0__Bacteria;D_1__Tenericutes;D_2__Mollicutes;D_3__Mycoplasmatales;D_4__Mycoplasmataceae;D_5__Mycoplasma;D_6__Mycoplasma parvum str. Indiana

Unassigned;__;__;__;__;__;__

D_0__Bacteria;D_1__Proteobacteria;D_2__Alphaproteobacteria;D_3__Rhizobiales;D_4__Beijerinckiaceae;D_5__Methylobacterium;D_6__Methylobacterium komagatae

D_0__Bacteria;D_1__Firmicutes;D_2__Bacilli;D_3__Lactobacillales;D_4__Lactobacillaceae;D_5__Lactobacillus;__

D_0__Bacteria;D_1__Patescibacteria;D_2__Parcubacteria;__;__;__;__

D_0__Bacteria;D_1__Proteobacteria;D_2__Alphaproteobacteria;D_3__Rhizobiales;D_4__Rhizobiaceae;D_5__Allorhizobium-Neorhizobium-Pararhizobium-Rhizobium;__

D_0__Bacteria;D_1__Spirochaetes;D_2__Spirochaetia;D_3__Spirochaetales;D_4__Spirochaetaceae;D_5__uncultured;D_6__Spirochaetaceae bacterium SURF-1

D_0__Bacteria;D_1__Proteobacteria;D_2__Gammaproteobacteria;D_3__Pseudomonadales;D_4__Pseudomonadaceae;D_5__Pseudomonas;__

D_0__Bacteria;D_1__Proteobacteria;D_2__Alphaproteobacteria;D_3__Rhodospirillales;D_4__Magnetospirillaceae;D_5__Magnetospirillum;__

D_0__Bacteria;D_1__Firmicutes;D_2__Negativicutes;D_3__Selenomonadales;D_4__Veillonellaceae;D_5__Megasphaera;D_6__Megasphaera elsdenii

D_0__Bacteria;D_1__Proteobacteria;D_2__Gammaproteobacteria;D_3__Pseudomonadales;D_4__Moraxellaceae;D_5__Acinetobacter;__

D_0__Bacteria;D_1__Patescibacteria;D_2__Parcubacteria;D_3__Candidatus Kaiserbacteria;D_4__uncultured bacterium;D_5__uncultured bacterium;D_6__uncultured bacterium

D_0__Bacteria;D_1__Proteobacteria;D_2__Alphaproteobacteria;D_3__Rhizobiales;D_4__Rhizobiaceae;D_5__Ochrobactrum;__

D_0__Bacteria;D_1__Firmicutes;D_2__Erysipelotrichia;D_3__Erysipelotrichales;D_4__Erysipelotrichaceae;D_5__Turicibacter;__

D_0__Bacteria;D_1__Chloroflexi;D_2__Anaerolineae;__;__;__;__

D_0__Bacteria;D_1__Firmicutes;D_2__Clostridia;D_3__Clostridiales;D_4__Peptostreptococcaceae;D_5__Romboutsia;__

D_0__Bacteria;D_1__Firmicutes;D_2__Clostridia;D_3__Clostridiales;D_4__Clostridiaceae 1;D_5__Clostridium sensu stricto 1;__

D_0__Bacteria;D_1__Proteobacteria;D_2__Gammaproteobacteria;D_3__Betaproteobacteriales;D_4__Burkholderiaceae;D_5__Curvibacter;__

D_0__Bacteria;D_1__Proteobacteria;D_2__Gammaproteobacteria;D_3__Betaproteobacteriales;D_4__Rhodocyclaceae;D_5__Denitratisoma;__

D_0__Bacteria;D_1__Cyanobacteria;D_2__Oxyphotobacteria;D_3__Chloroplast;__;__;__

D_0__Bacteria;D_1__Proteobacteria;D_2__Alphaproteobacteria;D_3__Rhodospirillales;D_4__uncultured;__;__

D_0__Bacteria;D_1__Patescibacteria;D_2__Parcubacteria;D_3__Candidatus Adlerbacteria;D_4__uncultured bacterium;D_5__uncultured bacterium;D_6__uncultured bacterium

D_0__Bacteria;D_1__Proteobacteria;D_2__Gammaproteobacteria;D_3__Betaproteobacteriales;D_4__TRA3-20;__;__

D_0__Bacteria;D_1__Proteobacteria;D_2__Gammaproteobacteria;D_3__Pseudomonadales;D_4__Moraxellaceae;D_5__Enhydrobacter;__

D_0__Bacteria;D_1__Firmicutes;D_2__Bacilli;D_3__Bacillales;D_4__Staphylococcaceae;D_5__Staphylococcus;__

D_0__Bacteria;D_1__Proteobacteria;D_2__Gammaproteobacteria;D_3__Betaproteobacteriales;D_4__Burkholderiaceae;D_5__Massilia;__

D_0__Bacteria;D_1__Firmicutes;D_2__Bacilli;D_3__Lactobacillales;D_4__Aerococcaceae;D_5__Ignavigranum;D_6__uncultured bacterium

D_0__Bacteria;D_1__Proteobacteria;D_2__Gammaproteobacteria;D_3__Xanthomonadales;D_4__Xanthomonadaceae;D_5__Stenotrophomonas;__

D_0__Bacteria;D_1__Firmicutes;D_2__Clostridia;D_3__Clostridiales;D_4__Christensenellaceae;D_5__Christensenellaceae R-7 group;D_6__uncultured organism

D_0__Bacteria;D_1__Firmicutes;D_2__Bacilli;D_3__Bacillales;D_4__Planococcaceae;D_5__Lysinibacillus;__

D_0__Bacteria;D_1__Proteobacteria;D_2__Alphaproteobacteria;D_3__Rhizobiales;D_4__Xanthobacteraceae;D_5__Bradyrhizobium;__

D_0__Bacteria;D_1__Firmicutes;D_2__Negativicutes;D_3__Selenomonadales;D_4__Veillonellaceae;D_5__Dialister;D_6__gut metagenome

D_0__Bacteria;D_1__Proteobacteria;D_2__Gammaproteobacteria;D_3__Pseudomonadales;D_4__Moraxellaceae;D_5__Moraxella;__

D_0__Bacteria;D_1__Proteobacteria;D_2__Gammaproteobacteria;D_3__Betaproteobacteriales;D_4__Burkholderiaceae;D_5__Herbaspirillum;__

D_0__Bacteria;D_1__Proteobacteria;D_2__Alphaproteobacteria;D_3__Rhizobiales;D_4__Rhizobiaceae;D_5__Mesorhizobium;__

D_0__Bacteria;D_1__Proteobacteria;D_2__Gammaproteobacteria;D_3__Pseudomonadales;D_4__Moraxellaceae;D_5__Acinetobacter;D_6__Acinetobacter lwoffii

D_0__Bacteria;D_1__Firmicutes;D_2__Bacilli;D_3__Lactobacillales;D_4__Aerococcaceae;D_5__Facklamia;D_6__uncultured Facklamia sp.

D_0__Bacteria;D_1__Firmicutes;D_2__Negativicutes;D_3__Selenomonadales;D_4__Veillonellaceae;D_5__uncultured;__

D_0__Bacteria;D_1__Proteobacteria;D_2__Gammaproteobacteria;D_3__Enterobacteriales;D_4__Enterobacteriaceae;__;__

D_0__Bacteria;D_1__Firmicutes;D_2__Bacilli;D_3__Lactobacillales;D_4__Carnobacteriaceae;D_5__Jeotgalibaca;__

D_0__Bacteria;D_1__Firmicutes;D_2__Bacilli;D_3__Lactobacillales;D_4__Enterococcaceae;D_5__Enterococcus;__

D_0__Bacteria;D_1__Patescibacteria;D_2__ABY1;D_3__Candidatus Magasanikbacteria;D_4__uncultured bacterium;D_5__uncultured bacterium;D_6__uncultured bacterium

D_0__Bacteria;D_1__Firmicutes;D_2__Negativicutes;D_3__Selenomonadales;D_4__Veillonellaceae;D_5__Megasphaera;__

D_0__Bacteria;D_1__Bacteroidetes;D_2__Bacteroidia;D_3__Chitinophagales;D_4__Chitinophagaceae;D_5__Ferruginibacter;__

D_0__Bacteria;D_1__Firmicutes;D_2__Bacilli;D_3__Bacillales;D_4__Bacillaceae;D_5__Bacillus;__

D_0__Bacteria;D_1__Bacteroidetes;D_2__Bacteroidia;D_3__Bacteroidales;D_4__Prevotellaceae;D_5__Prevotella 7;D_6__uncultured bacterium

D_0__Bacteria;D_1__Bacteroidetes;D_2__Bacteroidia;D_3__Chitinophagales;D_4__Saprospiraceae;D_5__uncultured;D_6__bacterium enrichment culture clone SRAO_34

D_0__Bacteria;D_1__Cyanobacteria;D_2__Oxyphotobacteria;D_3__Chloroplast;D_4__Phaseolus acutifolius (tepary bean);D_5__Phaseolus acutifolius (tepary bean);D_6__Phaseolus acutifolius (tepary bean)

D_0__Bacteria;D_1__Firmicutes;D_2__Clostridia;D_3__Clostridiales;D_4__Lachnospiraceae;D_5__Roseburia;__

D_0__Bacteria;D_1__Bacteroidetes;D_2__Bacteroidia;D_3__Chitinophagales;D_4__Chitinophagaceae;D_5__Sediminibacterium;D_6__mine drainage metagenome

D_0__Bacteria;D_1__Firmicutes;D_2__Negativicutes;D_3__Selenomonadales;D_4__Veillonellaceae;D_5__uncultured;D_6__uncultured rumen bacterium

D_0__Bacteria;D_1__Proteobacteria;D_2__Gammaproteobacteria;D_3__Betaproteobacteriales;D_4__Burkholderiaceae;__;__

D_0__Bacteria;D_1__Firmicutes;D_2__Clostridia;D_3__Clostridiales;D_4__Clostridiaceae 1;D_5__Clostridium sensu stricto 6;D_6__Clostridium bornimense

D_0__Bacteria;D_1__Proteobacteria;D_2__Alphaproteobacteria;D_3__Sphingomonadales;D_4__Sphingomonadaceae;D_5__Sphingomonas;__

D_0__Bacteria;D_1__Firmicutes;D_2__Negativicutes;D_3__Selenomonadales;D_4__Veillonellaceae;D_5__Mitsuokella;D_6__uncultured bacterium

D_0__Bacteria;D_1__Actinobacteria;D_2__Actinobacteria;D_3__Corynebacteriales;D_4__Corynebacteriaceae;D_5__Corynebacterium 1;__

D_0__Bacteria;D_1__Tenericutes;D_2__Mollicutes;D_3__Mycoplasmatales;D_4__Mycoplasmataceae;D_5__Mycoplasma;D_6__Mycoplasma flocculare

D_0__Bacteria;D_1__Proteobacteria;D_2__Gammaproteobacteria;D_3__Betaproteobacteriales;D_4__Burkholderiaceae;D_5__Bordetella;D_6__Bordetella pertussis

D_0__Bacteria;D_1__Firmicutes;D_2__Bacilli;D_3__Lactobacillales;D_4__Aerococcaceae;D_5__Aerosphaera;D_6__uncultured bacterium

D_0__Bacteria;D_1__Actinobacteria;D_2__Actinobacteria;D_3__Corynebacteriales;D_4__Nocardiaceae;D_5__Rhodococcus;__

D_0__Bacteria;D_1__Planctomycetes;D_2__Brocadiae;D_3__Brocadiales;D_4__Brocadiaceae;D_5__Candidatus Brocadia;D_6__Planctomycetes bacterium RIFOXYD12_FULL_42_12

D_0__Bacteria;D_1__Actinobacteria;D_2__Actinobacteria;D_3__Bifidobacteriales;D_4__Bifidobacteriaceae;D_5__Bifidobacterium;__

D_0__Bacteria;D_1__Gemmatimonadetes;D_2__Gemmatimonadetes;D_3__Gemmatimonadales;D_4__Gemmatimonadaceae;D_5__uncultured;

D_0__Bacteria;D_1__Gemmatimonadetes;D_2__Gemmatimonadetes;D_3__Gemmatimonadales;D_4__Gemmatimonadaceae;D_5__uncultured;__

D_0__Bacteria;D_1__Cyanobacteria;D_2__Melainabacteria;D_3__Obscuribacterales;D_4__uncultured bacterium;D_5__uncultured bacterium;D_6__uncultured bacterium

D_0__Bacteria;D_1__Firmicutes;D_2__Clostridia;D_3__Clostridiales;D_4__Lachnospiraceae;D_5__Agathobacter;__

D_0__Bacteria;D_1__Proteobacteria;D_2__Gammaproteobacteria;D_3__Betaproteobacteriales;D_4__Burkholderiaceae;D_5__Herbaspirillum;D_6__Herbaspirillum huttiense subsp. huttiense

D_0__Bacteria;D_1__Bacteroidetes;D_2__Bacteroidia;D_3__Flavobacteriales;D_4__Weeksellaceae;D_5__Bergeyella;D_6__Bergeyella porcorum

D_0__Bacteria;D_1__Firmicutes;D_2__Clostridia;D_3__Clostridiales;D_4__Lachnospiraceae;D_5__Syntrophococcus;D_6__uncultured bacterium

D_0__Bacteria;D_1__Bacteroidetes;D_2__Bacteroidia;D_3__Bacteroidales;D_4__Muribaculaceae;D_5__uncultured Porphyromonadaceae bacterium;D_6__uncultured Porphyromonadaceae bacterium

D_0__Bacteria;D_1__Actinobacteria;D_2__Actinobacteria;D_3__Propionibacteriales;D_4__Propionibacteriaceae;D_5__Cutibacterium;__

D_0__Bacteria;D_1__Firmicutes;D_2__Clostridia;D_3__Clostridiales;D_4__Ruminococcaceae;D_5__[Eubacterium] coprostanoligenes group;D_6__uncultured organism

D_0__Bacteria;D_1__Firmicutes;D_2__Negativicutes;D_3__Selenomonadales;D_4__Veillonellaceae;D_5__Selenomonas;D_6__uncultured rumen bacterium

D_0__Bacteria;D_1__Firmicutes;D_2__Clostridia;D_3__Clostridiales;D_4__Ruminococcaceae;D_5__Subdoligranulum;__

D_0__Bacteria;D_1__Proteobacteria;D_2__Alphaproteobacteria;D_3__Rhizobiales;D_4__Hyphomicrobiaceae;D_5__Pedomicrobium;__

D_0__Bacteria;D_1__Firmicutes;D_2__Clostridia;D_3__Clostridiales;D_4__Christensenellaceae;D_5__Christensenellaceae R-7 group;D_6__uncultured spirochete

D_0__Bacteria;D_1__Bacteroidetes;D_2__Bacteroidia;D_3__Bacteroidales;D_4__Prevotellaceae;D_5__Prevotella 9;__

D_0__Bacteria;D_1__Proteobacteria;D_2__Gammaproteobacteria;D_3__Pasteurellales;D_4__Pasteurellaceae;D_5__Actinobacillus;D_6__[Haemophilus] parasuis

D_0__Bacteria;D_1__Firmicutes;D_2__Clostridia;D_3__Clostridiales;D_4__Ruminococcaceae;D_5__Ruminococcaceae NK4A214 group;D_6__metagenome

D_0__Bacteria;D_1__Firmicutes;D_2__Clostridia;D_3__Clostridiales;D_4__Ruminococcaceae;D_5__uncultured;D_6__Ruminococcus sp. YE281

D_0__Bacteria;D_1__Bacteroidetes;D_2__Bacteroidia;D_3__Bacteroidales;D_4__Prevotellaceae;__;__

D_0__Bacteria;D_1__Firmicutes;D_2__Bacilli;D_3__Bacillales;D_4__Family XII;D_5__Exiguobacterium;__

D_0__Bacteria;D_1__Firmicutes;D_2__Clostridia;D_3__Clostridiales;D_4__Ruminococcaceae;D_5__Ruminococcaceae UCG-014;__

D_0__Bacteria;D_1__Bacteroidetes;D_2__Bacteroidia;D_3__Flavobacteriales;D_4__Weeksellaceae;D_5__Chryseobacterium;__

D_0__Bacteria;D_1__Firmicutes;D_2__Clostridia;D_3__Clostridiales;D_4__Ruminococcaceae;D_5__Ruminococcaceae UCG-005;__

D_0__Bacteria;D_1__Firmicutes;D_2__Negativicutes;D_3__Selenomonadales;D_4__Veillonellaceae;D_5__Anaerospora;D_6__uncultured bacterium

D_0__Bacteria;D_1__Firmicutes;D_2__Bacilli;D_3__Lactobacillales;D_4__Enterococcaceae;D_5__Vagococcus;D_6__Vagococcus fluvialis

D_0__Bacteria;D_1__Proteobacteria;D_2__Alphaproteobacteria;D_3__Reyranellales;D_4__Reyranellaceae;D_5__Reyranella;__

D_0__Bacteria;D_1__Firmicutes;D_2__Bacilli;D_3__Lactobacillales;D_4__Carnobacteriaceae;D_5__Jeotgalibaca;D_6__uncultured bacterium

D_0__Bacteria;D_1__Proteobacteria;D_2__Alphaproteobacteria;D_3__Dongiales;D_4__Dongiaceae;D_5__Dongia;__

D_0__Bacteria;D_1__Proteobacteria;D_2__Deltaproteobacteria;D_3__Myxococcales;D_4__mle1-27;D_5__uncultured bacterium;D_6__uncultured bacterium

D_0__Bacteria;D_1__Firmicutes;D_2__Clostridia;D_3__Clostridiales;D_4__Ruminococcaceae;D_5__Ruminococcus 1;__

D_0__Bacteria;D_1__Bacteroidetes;D_2__Bacteroidia;D_3__Bacteroidales;D_4__Rikenellaceae;D_5__Rikenellaceae RC9 gut group;D_6__uncultured bacterium

D_0__Bacteria;D_1__Firmicutes;D_2__Clostridia;D_3__Clostridiales;D_4__Ruminococcaceae;D_5__Ruminococcaceae UCG-014;D_6__uncultured bacterium

D_0__Bacteria;D_1__Proteobacteria;D_2__Gammaproteobacteria;D_3__Aeromonadales;D_4__Succinivibrionaceae;D_5__Succinivibrio;__

D_0__Bacteria;D_1__Firmicutes;D_2__Bacilli;D_3__Lactobacillales;D_4__Carnobacteriaceae;D_5__uncultured;D_6__uncultured bacterium

D_0__Bacteria;D_1__Bacteroidetes;D_2__Bacteroidia;D_3__Bacteroidales;D_4__Bacteroidaceae;D_5__Bacteroides;D_6__uncultured bacterium

D_0__Bacteria;D_1__Bacteroidetes;D_2__Bacteroidia;D_3__Bacteroidales;D_4__Prevotellaceae;D_5__Prevotella 7;D_6__Prevotella sp. RS2

D_0__Bacteria;D_1__Proteobacteria;D_2__Deltaproteobacteria;D_3__Myxococcales;D_4__mle1-27;D_5__uncultured Myxococcales bacterium;D_6__uncultured Myxococcales bacterium

D_0__Bacteria;D_1__Firmicutes;D_2__Clostridia;D_3__Clostridiales;D_4__Ruminococcaceae;D_5__Ruminococcus 2;D_6__uncultured bacterium

D_0__Bacteria;D_1__Firmicutes;D_2__Negativicutes;D_3__Selenomonadales;D_4__Acidaminococcaceae;D_5__Phascolarctobacterium;__

D_0__Bacteria;D_1__Bacteroidetes;D_2__Bacteroidia;D_3__Bacteroidales;D_4__Muribaculaceae;__;__

D_0__Bacteria;D_1__Proteobacteria;D_2__Alphaproteobacteria;D_3__Rickettsiales;D_4__Mitochondria;__;__

D_0__Bacteria;D_1__Proteobacteria;D_2__Gammaproteobacteria;D_3__Betaproteobacteriales;D_4__Rhodocyclaceae;D_5__Azospira;__

D_0__Archaea;D_1__Diapherotrites;D_2__Micrarchaeia;D_3__uncultured archaeon;D_4__uncultured archaeon;D_5__uncultured archaeon;D_6__uncultured archaeon

D_0__Bacteria;D_1__Bacteroidetes;D_2__Ignavibacteria;D_3__Ignavibacteriales;D_4__SR-FBR-L83;D_5__uncultured bacterium;D_6__uncultured bacterium

D_0__Bacteria;D_1__Tenericutes;D_2__Mollicutes;D_3__Mollicutes RF39;__;__;__

D_0__Bacteria;D_1__Firmicutes;D_2__Clostridia;D_3__Clostridiales;D_4__Family XIII;D_5__Family XIII AD3011 group;D_6__uncultured organism

D_0__Bacteria;D_1__Chlamydiae;D_2__Chlamydiae;D_3__Chlamydiales;D_4__Simkaniaceae;D_5__uncultured;__

D_0__Bacteria;D_1__Firmicutes;D_2__Bacilli;D_3__Lactobacillales;D_4__Lactobacillaceae;D_5__Lactobacillus;D_6__Lactobacillus ruminis

D_0__Bacteria;D_1__Firmicutes;D_2__Negativicutes;D_3__Selenomonadales;D_4__Veillonellaceae;D_5__Veillonella;__

D_0__Bacteria;D_1__Firmicutes;D_2__Clostridia;D_3__Clostridiales;D_4__Ruminococcaceae;D_5__Ruminococcaceae UCG-008;D_6__uncultured bacterium

D_0__Bacteria;D_1__Firmicutes;D_2__Clostridia;D_3__Clostridiales;D_4__Eubacteriaceae;D_5__Pseudoramibacter;D_6__uncultured bacterium

D_0__Bacteria;D_1__Acidobacteria;D_2__Holophagae;D_3__Subgroup 7;__;__;__

D_0__Bacteria;D_1__Bacteroidetes;D_2__Bacteroidia;D_3__Bacteroidales;D_4__Prevotellaceae;D_5__Prevotella 9;D_6__uncultured bacterium

D_0__Bacteria;D_1__Proteobacteria;D_2__Alphaproteobacteria;__;__;__;__

D_0__Bacteria;D_1__Firmicutes;D_2__Negativicutes;D_3__Selenomonadales;D_4__Acidaminococcaceae;D_5__Acidaminococcus;__

D_0__Bacteria;D_1__Patescibacteria;D_2__ABY1;D_3__Candidatus Magasanikbacteria;D_4__Candidatus Magasanikbacteria bacterium RIFCSPHIGHO2_02_FULL_50_9b;D_5__Candidatus Magasanikbacteria bacterium RIFCSPHIGH

D_0__Bacteria;D_1__Firmicutes;D_2__Erysipelotrichia;D_3__Erysipelotrichales;D_4__Erysipelotrichaceae;D_5__Catenibacterium;__

D_0__Bacteria;D_1__Firmicutes;D_2__Bacilli;D_3__Lactobacillales;D_4__Streptococcaceae;D_5__Lactococcus;__

D_0__Bacteria;D_1__Proteobacteria;D_2__Gammaproteobacteria;D_3__Enterobacteriales;D_4__Enterobacteriaceae;D_5__Klebsiella;__

D_0__Bacteria;D_1__Proteobacteria;D_2__Gammaproteobacteria;D_3__Betaproteobacteriales;D_4__Burkholderiaceae;D_5__Achromobacter;__

D_0__Bacteria;D_1__Firmicutes;D_2__Clostridia;D_3__Clostridiales;D_4__Ruminococcaceae;D_5__Faecalibacterium;__

D_0__Bacteria;D_1__Actinobacteria;D_2__Coriobacteriia;D_3__Coriobacteriales;D_4__Atopobiaceae;D_5__Olsenella;D_6__metagenome

D_0__Bacteria;D_1__Firmicutes;D_2__Clostridia;D_3__Clostridiales;D_4__Ruminococcaceae;D_5__Ruminococcaceae UCG-005;D_6__uncultured organism

D_0__Bacteria;D_1__Proteobacteria;D_2__Gammaproteobacteria;D_3__WD260;D_4__uncultured bacterium;D_5__uncultured bacterium;D_6__uncultured bacterium

D_0__Bacteria;D_1__Firmicutes;D_2__Clostridia;D_3__Clostridiales;D_4__Ruminococcaceae;D_5__Intestinimonas;D_6__uncultured bacterium

D_0__Bacteria;D_1__Firmicutes;D_2__Erysipelotrichia;D_3__Erysipelotrichales;D_4__Erysipelotrichaceae;D_5__uncultured;D_6__uncultured bacterium

D_0__Bacteria;D_1__Verrucomicrobia;D_2__Verrucomicrobiae;D_3__Pedosphaerales;D_4__Pedosphaeraceae;D_5__uncultured bacterium;D_6__uncultured bacterium

D_0__Bacteria;D_1__Proteobacteria;D_2__Gammaproteobacteria;D_3__Betaproteobacteriales;D_4__Burkholderiaceae;D_5__Noviherbaspirillum;__

D_0__Bacteria;D_1__Firmicutes;D_2__Negativicutes;D_3__Selenomonadales;D_4__Veillonellaceae;D_5__uncultured;D_6__uncultured rumen bacterium 3C28d-7

D_0__Bacteria;D_1__Proteobacteria;D_2__Gammaproteobacteria;D_3__Betaproteobacteriales;D_4__Burkholderiaceae;D_5__Curvibacter;D_6__uncultured beta proteobacterium

D_0__Bacteria;D_1__Firmicutes;D_2__Clostridia;D_3__Clostridiales;D_4__Lachnospiraceae;D_5__[Ruminococcus] gauvreauii group;__

D_0__Bacteria;D_1__Firmicutes;D_2__Clostridia;D_3__Clostridiales;D_4__Family XIII;D_5__Family XIII AD3011 group;D_6__uncultured bacterium

D_0__Bacteria;D_1__Firmicutes;D_2__Clostridia;D_3__Clostridiales;D_4__Lachnospiraceae;D_5__Blautia;__

D_0__Bacteria;D_1__Patescibacteria;D_2__Gracilibacteria;D_3__Candidatus Abawacabacteria;D_4__Candidatus Abawacabacteria bacterium RBG_16_42_10;D_5__Candidatus Abawacabacteria bacterium RBG_16_42_10;D_6__Candi

D_0__Bacteria;D_1__Firmicutes;D_2__Negativicutes;D_3__Selenomonadales;D_4__Acidaminococcaceae;D_5__Succiniclasticum;D_6__uncultured rumen bacterium 4C28d-16

D_0__Archaea;D_1__Euryarchaeota;D_2__Methanobacteria;D_3__Methanobacteriales;D_4__Methanobacteriaceae;D_5__Methanobrevibacter;D_6__uncultured Methanobrevibacter sp.

D_0__Bacteria;D_1__Firmicutes;D_2__Clostridia;D_3__Clostridiales;D_4__Ruminococcaceae;D_5__[Eubacterium] coprostanoligenes group;__

D_0__Bacteria;D_1__Firmicutes;D_2__Bacilli;D_3__Lactobacillales;D_4__Carnobacteriaceae;D_5__Atopostipes;D_6__uncultured bacterium

D_0__Bacteria;D_1__Firmicutes;D_2__Clostridia;D_3__Clostridiales;D_4__Lachnospiraceae;D_5__Lachnospiraceae NK4A136 group;__

D_0__Bacteria;D_1__Firmicutes;D_2__Clostridia;D_3__Clostridiales;D_4__Ruminococcaceae;D_5__Ruminococcaceae UCG-002;__

D_0__Bacteria;D_1__Firmicutes;D_2__Bacilli;D_3__Lactobacillales;D_4__Lactobacillaceae;D_5__Lactobacillus;D_6__Lactobacillus nagelii DSM 13675

D_0__Bacteria;D_1__Firmicutes;D_2__Negativicutes;D_3__Selenomonadales;D_4__Veillonellaceae;D_5__Anaerovibrio;__

D_0__Bacteria;D_1__Proteobacteria;D_2__Gammaproteobacteria;D_3__Pasteurellales;D_4__Pasteurellaceae;D_5__Actinobacillus;D_6__Actinobacillus indolicus

D_0__Bacteria;D_1__Firmicutes;D_2__Negativicutes;D_3__Selenomonadales;D_4__Veillonellaceae;D_5__Mitsuokella;__

D_0__Bacteria;D_1__Firmicutes;D_2__Negativicutes;D_3__Selenomonadales;D_4__Veillonellaceae;D_5__Selenomonas;D_6__bacterium mpn-isolate group 9

D_0__Bacteria;D_1__Omnitrophicaeota;__;__;__;__;__

D_0__Bacteria;D_1__Bacteroidetes;D_2__Bacteroidia;D_3__Chitinophagales;D_4__Chitinophagaceae;D_5__Terrimonas;__

D_0__Bacteria;D_1__Spirochaetes;D_2__Spirochaetia;D_3__Spirochaetales;D_4__Spirochaetaceae;D_5__Treponema 2;D_6__Treponema succinifaciens DSM 2489

D_0__Bacteria;D_1__Firmicutes;D_2__Bacilli;D_3__Lactobacillales;D_4__Lactobacillaceae;D_5__Lactobacillus;D_6__Lactobacillus reuteri

D_0__Bacteria;D_1__Firmicutes;D_2__Bacilli;D_3__Lactobacillales;D_4__Carnobacteriaceae;D_5__Lacticigenium;D_6__uncultured bacterium

D_0__Bacteria;D_1__Firmicutes;D_2__Bacilli;D_3__Lactobacillales;D_4__Leuconostocaceae;D_5__Weissella;D_6__Weissella paramesenteroides

D_0__Bacteria;D_1__Firmicutes;D_2__Clostridia;D_3__Clostridiales;D_4__Lachnospiraceae;__;__

D_0__Bacteria;D_1__Proteobacteria;D_2__Alphaproteobacteria;D_3__Rhodospirillales;D_4__uncultured;D_5__uncultured Rhodospirillaceae bacterium;D_6__uncultured Rhodospirillaceae bacterium

D_0__Bacteria;D_1__Bacteroidetes;D_2__Bacteroidia;D_3__Flavobacteriales;D_4__Weeksellaceae;D_5__Chryseobacterium;D_6__Chryseobacterium taklimakanense

D_0__Bacteria;D_1__Firmicutes;D_2__Clostridia;D_3__Clostridiales;D_4__Lachnospiraceae;D_5__[Eubacterium] hallii group;D_6__uncultured organism

D_0__Bacteria;D_1__Proteobacteria;D_2__Gammaproteobacteria;D_3__Pseudomonadales;D_4__Moraxellaceae;D_5__Psychrobacter;__

D_0__Bacteria;D_1__Proteobacteria;D_2__Alphaproteobacteria;D_3__Rhizobiales;D_4__Xanthobacteraceae;D_5__Rhodoplanes;__

D_0__Bacteria;D_1__Acidobacteria;D_2__Acidobacteriia;D_3__Acidobacteriales;D_4__uncultured;__;__

D_0__Bacteria;D_1__Proteobacteria;D_2__Gammaproteobacteria;D_3__Legionellales;D_4__Legionellaceae;D_5__Legionella;__

D_0__Bacteria;D_1__Proteobacteria;D_2__Gammaproteobacteria;D_3__Betaproteobacteriales;D_4__Burkholderiaceae;D_5__Ramlibacter;__

D_0__Bacteria;D_1__Proteobacteria;D_2__Gammaproteobacteria;D_3__Pasteurellales;D_4__Pasteurellaceae;D_5__Actinobacillus;D_6__Actinobacillus minor

D_0__Bacteria;D_1__Patescibacteria;D_2__Gracilibacteria;D_3__Candidatus Peregrinibacteria;D_4__uncultured bacterium;D_5__uncultured bacterium;D_6__uncultured bacterium

D_0__Bacteria;D_1__Firmicutes;D_2__Clostridia;D_3__Clostridiales;D_4__Christensenellaceae;D_5__Christensenellaceae R-7 group;D_6__gut metagenome

D_0__Bacteria;D_1__Bacteroidetes;D_2__Ignavibacteria;D_3__SJA-28;D_4__uncultured Chlorobi bacterium;D_5__uncultured Chlorobi bacterium;D_6__uncultured Chlorobi bacterium

D_0__Bacteria;D_1__Proteobacteria;D_2__Deltaproteobacteria;D_3__Oligoflexales;D_4__0319-6G20;D_5__uncultured Roseobacter sp.;D_6__uncultured Roseobacter sp.

D_0__Bacteria;D_1__Bacteroidetes;D_2__Bacteroidia;D_3__Bacteroidales;D_4__Prevotellaceae;D_5__Alloprevotella;D_6__uncultured bacterium

D_0__Bacteria;D_1__Bacteroidetes;D_2__Bacteroidia;D_3__Bacteroidales;D_4__Prevotellaceae;D_5__Prevotellaceae NK3B31 group;D_6__uncultured bacterium

D_0__Bacteria;D_1__Acidobacteria;D_2__Subgroup 6;D_3__uncultured microorganism;D_4__uncultured microorganism;D_5__uncultured microorganism;D_6__uncultured microorganism

D_0__Bacteria;D_1__Proteobacteria;D_2__Gammaproteobacteria;D_3__Betaproteobacteriales;D_4__Burkholderiaceae;D_5__Polynucleobacter;D_6__Polynucleobacter cosmopolitanus

D_0__Bacteria;D_1__Bacteroidetes;D_2__Bacteroidia;D_3__Bacteroidales;D_4__Porphyromonadaceae;D_5__Porphyromonas;D_6__Porphyromonas sp. feline oral taxon 109

D_0__Bacteria;D_1__Firmicutes;D_2__Clostridia;D_3__Clostridiales;D_4__Ruminococcaceae;D_5__Ruminococcaceae UCG-005;D_6__uncultured bacterium

D_0__Bacteria;D_1__Proteobacteria;D_2__Gammaproteobacteria;D_3__Pseudomonadales;D_4__Moraxellaceae;D_5__Acinetobacter;D_6__Acinetobacter guillouiae

D_0__Bacteria;D_1__Tenericutes;D_2__Mollicutes;D_3__Mycoplasmatales;D_4__Mycoplasmataceae;D_5__Mycoplasma;D_6__Mycoplasma coccoides

D_0__Bacteria;D_1__Firmicutes;D_2__Bacilli;D_3__Lactobacillales;D_4__Aerococcaceae;D_5__Facklamia;__

D_0__Bacteria;D_1__Proteobacteria;D_2__Alphaproteobacteria;D_3__Rhodobacterales;D_4__Rhodobacteraceae;D_5__Gemmobacter;__

D_0__Bacteria;D_1__Proteobacteria;D_2__Deltaproteobacteria;D_3__Syntrophobacterales;D_4__Syntrophaceae;D_5__Smithella;__

D_0__Bacteria;D_1__Patescibacteria;D_2__Parcubacteria;D_3__Candidatus Nomurabacteria;D_4__uncultured Parcubacteria group bacterium;D_5__uncultured Parcubacteria group bacterium;D_6__uncultured Parcubacteria group bacte

D_0__Bacteria;D_1__Proteobacteria;D_2__Alphaproteobacteria;D_3__Sphingomonadales;D_4__Sphingomonadaceae;__;__

D_0__Bacteria;D_1__Epsilonbacteraeota;D_2__Campylobacteria;D_3__Campylobacterales;D_4__Arcobacteraceae;D_5__Arcobacter;__

D_0__Bacteria;D_1__Bacteroidetes;D_2__Ignavibacteria;D_3__Kryptoniales;D_4__BSV26;D_5__uncultured green sulfur bacterium GR-296.II.73;D_6__uncultured green sulfur bacterium GR-296.II.73

D_0__Bacteria;D_1__Firmicutes;D_2__Bacilli;D_3__Bacillales;D_4__Bacillaceae;D_5__Anoxybacillus;__

D_0__Bacteria;D_1__Firmicutes;D_2__Erysipelotrichia;D_3__Erysipelotrichales;D_4__Erysipelotrichaceae;D_5__Solobacterium;D_6__uncultured Bulleidia sp.

D_0__Bacteria;D_1__Firmicutes;D_2__Clostridia;D_3__Clostridiales;D_4__Peptostreptococcaceae;D_5__Terrisporobacter;__

D_0__Bacteria;D_1__Proteobacteria;D_2__Deltaproteobacteria;D_3__Myxococcales;D_4__Polyangiaceae;D_5__Pajaroellobacter;__

D_0__Bacteria;D_1__Patescibacteria;D_2__Saccharimonadia;D_3__Saccharimonadales;__;__;__

D_0__Bacteria;D_1__Proteobacteria;D_2__Alphaproteobacteria;D_3__Rhizobiales;D_4__Xanthobacteraceae;__;__

D_0__Bacteria;D_1__Proteobacteria;D_2__Deltaproteobacteria;D_3__Myxococcales;D_4__Polyangiaceae;D_5__Pajaroellobacter;D_6__uncultured Polyangiaceae bacterium

D_0__Bacteria;D_1__Patescibacteria;D_2__Parcubacteria;D_3__uncultured bacterium;D_4__uncultured bacterium;D_5__uncultured bacterium;D_6__uncultured bacterium

D_0__Bacteria;D_1__Proteobacteria;D_2__Deltaproteobacteria;D_3__Myxococcales;D_4__bacteriap25;D_5__uncultured bacterium;D_6__uncultured bacterium

D_0__Bacteria;D_1__Proteobacteria;D_2__Gammaproteobacteria;D_3__Enterobacteriales;D_4__Enterobacteriaceae;D_5__Serratia;__

D_0__Bacteria;D_1__Firmicutes;D_2__Clostridia;D_3__Clostridiales;D_4__Lachnospiraceae;D_5__Anaerosporobacter;D_6__uncultured Lachnospiraceae bacterium

D_0__Bacteria;D_1__Cyanobacteria;D_2__Oxyphotobacteria;D_3__Chloroplast;D_4__Durinskia baltica;D_5__Durinskia baltica;D_6__Durinskia baltica

D_0__Bacteria;D_1__Bacteroidetes;D_2__Bacteroidia;D_3__Bacteroidales;D_4__Rikenellaceae;D_5__dgA-11 gut group;D_6__uncultured Bacteroidales bacterium

D_0__Bacteria;D_1__Proteobacteria;D_2__Gammaproteobacteria;D_3__Aeromonadales;D_4__Succinivibrionaceae;D_5__Succinivibrio;D_6__uncultured Succinivibrio sp.

D_0__Bacteria;D_1__Proteobacteria;D_2__Gammaproteobacteria;D_3__Betaproteobacteriales;D_4__Rhodocyclaceae;__;__

D_0__Bacteria;D_1__Proteobacteria;D_2__Alphaproteobacteria;D_3__Rhizobiales;D_4__Rhizobiales Incertae Sedis;D_5__uncultured;__

D_0__Bacteria;D_1__Proteobacteria;D_2__Gammaproteobacteria;D_3__Pseudomonadales;D_4__Pseudomonadaceae;D_5__Pseudomonas;D_6__Pseudomonas sp. MN11-3

D_0__Bacteria;D_1__Firmicutes;D_2__Bacilli;D_3__Lactobacillales;D_4__Enterococcaceae;D_5__Enterococcus;D_6__Enterococcus sp. MES2

D_0__Bacteria;D_1__Firmicutes;D_2__Bacilli;D_3__Lactobacillales;D_4__Carnobacteriaceae;D_5__Granulicatella;__

D_0__Bacteria;D_1__Firmicutes;D_2__Clostridia;D_3__Clostridiales;D_4__Lachnospiraceae;D_5__Oribacterium;D_6__uncultured bacterium

D_0__Bacteria;D_1__Proteobacteria;D_2__Gammaproteobacteria;D_3__Betaproteobacteriales;D_4__Rhodocyclaceae;D_5__Dechloromonas;__

D_0__Bacteria;D_1__Spirochaetes;D_2__Leptospirae;D_3__Leptospirales;D_4__Leptospiraceae;D_5__uncultured;D_6__uncultured bacterium

D_0__Bacteria;D_1__Proteobacteria;D_2__Alphaproteobacteria;D_3__Rhizobiales;D_4__Rhizobiales Incertae Sedis;D_5__uncultured;D_6__uncultured bacterium

D_0__Bacteria;D_1__Firmicutes;D_2__Clostridia;D_3__Clostridiales;D_4__Ruminococcaceae;D_5__Ruminiclostridium 5;__

D_0__Bacteria;D_1__Firmicutes;D_2__Clostridia;D_3__Clostridiales;D_4__Ruminococcaceae;D_5__Fastidiosipila;__

D_0__Bacteria;D_1__Bacteroidetes;D_2__Bacteroidia;D_3__Bacteroidales;D_4__Muribaculaceae;D_5__uncultured bacterium;D_6__uncultured bacterium

D_0__Bacteria;D_1__Patescibacteria;D_2__Parcubacteria;D_3__Candidatus Brennerbacteria;__;__;__

D_0__Bacteria;D_1__Firmicutes;D_2__Clostridia;D_3__Clostridiales;D_4__Ruminococcaceae;D_5__[Eubacterium] coprostanoligenes group;D_6__uncultured Clostridium sp.

D_0__Bacteria;D_1__Firmicutes;D_2__Clostridia;D_3__Clostridiales;D_4__Christensenellaceae;D_5__Christensenellaceae R-7 group;D_6__uncultured prokaryote

D_0__Bacteria;D_1__Proteobacteria;D_2__Gammaproteobacteria;D_3__Betaproteobacteriales;D_4__Rhodocyclaceae;D_5__Propionivibrio;D_6__uncultured bacterium

D_0__Bacteria;D_1__Acetothermia;D_2__Acetothermiia;D_3__uncultured Acetothermia bacterium;D_4__uncultured Acetothermia bacterium;D_5__uncultured Acetothermia bacterium;D_6__uncultured Acetothermia bacterium

D_0__Bacteria;D_1__Firmicutes;D_2__Clostridia;D_3__Clostridiales;D_4__Christensenellaceae;D_5__Christensenellaceae R-7 group;__

D_0__Bacteria;D_1__Firmicutes;D_2__Clostridia;D_3__Clostridiales;D_4__Ruminococcaceae;D_5__Ruminiclostridium 6;__

D_0__Bacteria;D_1__Proteobacteria;D_2__Gammaproteobacteria;D_3__Cellvibrionales;D_4__Cellvibrionaceae;D_5__uncultured;D_6__uncultured bacterium

D_0__Bacteria;D_1__Firmicutes;D_2__Clostridia;D_3__Clostridiales;D_4__Ruminococcaceae;D_5__Oscillospira;D_6__uncultured bacterium

D_0__Bacteria;D_1__Actinobacteria;D_2__Actinobacteria;D_3__Pseudonocardiales;D_4__Pseudonocardiaceae;D_5__Lechevalieria;__

D_0__Bacteria;D_1__Patescibacteria;D_2__Parcubacteria;D_3__Candidatus Nomurabacteria;D_4__uncultured soil bacterium;D_5__uncultured soil bacterium;D_6__uncultured soil bacterium

D_0__Bacteria;D_1__Firmicutes;D_2__Clostridia;D_3__Clostridiales;D_4__Ruminococcaceae;D_5__Ruminiclostridium;D_6__uncultured rumen bacterium

D_0__Bacteria;D_1__Firmicutes;D_2__Bacilli;D_3__Lactobacillales;D_4__Lactobacillaceae;D_5__Pediococcus;__
D_0__Bacteria;D_1__Proteobacteria;D_2__Gammaproteobacteria;D_3__Enterobacteriales;D_4__Enterobacteriaceae;D_5__Citrobacter;__
D_0__Bacteria;D_1__Firmicutes;D_2__Negativicutes;D_3__Selenomonadales;D_4__Veillonellaceae;__;__
D_0__Bacteria;D_1__Actinobacteria;D_2__Actinobacteria;D_3__PeM15;D_4__metagenome;D_5__metagenome;D_6__metagenome
D_0__Bacteria;D_1__Proteobacteria;D_2__Gammaproteobacteria;D_3__Betaproteobacteriales;D_4__Burkholderiaceae;D_5__Comamonas;D_6__Comamonas sp. S3 SY-2016
D_0__Bacteria;D_1__Proteobacteria;D_2__Alphaproteobacteria;D_3__Rhizobiales;D_4__Beijerinckiaceae;D_5__Bosea;__
D_0__Bacteria;D_1__Verrucomicrobia;D_2__Verrucomicrobiae;D_3__Verrucomicrobiales;D_4__Rubritaleaceae;D_5__Luteolibacter;__
D_0__Bacteria;D_1__Actinobacteria;D_2__Actinobacteria;D_3__Corynebacteriales;D_4__Corynebacteriaceae;D_5__Corynebacterium 1;D_6__Corynebacterium glutamicum
D_0__Bacteria;D_1__Bacteroidetes;D_2__Bacteroidia;D_3__Chitinophagales;D_4__Chitinophagaceae;D_5__Segetibacter;__
D_0__Bacteria;D_1__Bacteroidetes;D_2__Bacteroidia;D_3__Bacteroidales;D_4__Prevotellaceae;D_5__Alloprevotella;D_6__Prevotella sp. canine oral taxon 282
D_0__Bacteria;D_1__Proteobacteria;D_2__Gammaproteobacteria;D_3__Betaproteobacteriales;__;__;__
D_0__Bacteria;D_1__Chloroflexi;D_2__Anaerolineae;D_3__Anaerolineales;D_4__Anaerolineaceae;D_5__uncultured;__
D_0__Bacteria;D_1__Patescibacteria;D_2__Gracilibacteria;D_3__Candidatus Peribacteria;__;__;__
D_0__Bacteria;D_1__Verrucomicrobia;D_2__Verrucomicrobiae;D_3__Opitutales;D_4__Opitutaceae;D_5__Lacunisphaera;D_6__Opitutus sp. NVT1
D_0__Bacteria;D_1__Proteobacteria;D_2__Deltaproteobacteria;D_3__Oligoflexales;D_4__0319-6G20;D_5__uncultured bacterium;D_6__uncultured bacterium
D_0__Bacteria;D_1__Proteobacteria;D_2__Alphaproteobacteria;D_3__Paracaedibacterales;D_4__Paracaedibacteraceae;D_5__Candidatus Captivus;D_6__Candidatus Captivus acidiprotistae
D_0__Bacteria;D_1__Firmicutes;D_2__Bacilli;D_3__Bacillales;D_4__Planococcaceae;D_5__Chryseomicrobium;__
D_0__Bacteria;D_1__Proteobacteria;D_2__Gammaproteobacteria;D_3__Enterobacteriales;D_4__Enterobacteriaceae;D_5__Enterobacter;__
D_0__Bacteria;D_1__Patescibacteria;D_2__Parcubacteria;D_3__Candidatus Liptonbacteria;D_4__Parcubacteria group bacterium GW2011_GWA1_60_11;D_5__Parcubacteria group bacterium GW2011_GWA1_60_11;D_6__Parcubacter
D_0__Bacteria;D_1__Proteobacteria;D_2__Alphaproteobacteria;D_3__Caulobacterales;D_4__Caulobacteraceae;D_5__Brevundimonas;__
D_0__Bacteria;D_1__Firmicutes;D_2__Erysipelotrichia;D_3__Erysipelotrichales;D_4__Erysipelotrichaceae;D_5__Erysipelotrichaceae UCG-009;D_6__uncultured rumen bacterium
D_0__Bacteria;D_1__Proteobacteria;D_2__Gammaproteobacteria;D_3__Pseudomonadales;D_4__Moraxellaceae;D_5__Acinetobacter;D_6__Acinetobacter schindleri
D_0__Bacteria;D_1__Actinobacteria;D_2__Actinobacteria;D_3__Corynebacteriales;D_4__Corynebacteriaceae;D_5__Corynebacterium 1;D_6__Corynebacterium thomssenii
D_0__Bacteria;D_1__Actinobacteria;D_2__Actinobacteria;D_3__Micrococcales;D_4__Dermabacteraceae;D_5__Brachybacterium;__
D_0__Bacteria;D_1__Proteobacteria;D_2__Alphaproteobacteria;D_3__uncultured;D_4__uncultured bacterium;D_5__uncultured bacterium;D_6__uncultured bacterium
D_0__Bacteria;D_1__Firmicutes;D_2__Clostridia;D_3__Clostridiales;D_4__Ruminococcaceae;D_5__Ruminococcus 1;D_6__gut metagenome
D_0__Bacteria;D_1__Firmicutes;D_2__Erysipelotrichia;D_3__Erysipelotrichales;D_4__Erysipelotrichaceae;D_5__Erysipelotrichaceae UCG-006;D_6__uncultured bacterium
D_0__Bacteria;D_1__Bacteroidetes;D_2__Bacteroidia;D_3__Bacteroidales;D_4__Prevotellaceae;D_5__Prevotellaceae NK3B31 group;__
D_0__Bacteria;D_1__Firmicutes;D_2__Bacilli;D_3__Bacillales;D_4__Family XI;D_5__Gemella;__
D_0__Bacteria;D_1__Firmicutes;D_2__Erysipelotrichia;D_3__Erysipelotrichales;D_4__Erysipelotrichaceae;D_5__Catenisphaera;D_6__uncultured rumen bacterium
D_0__Bacteria;D_1__Bacteroidetes;D_2__Bacteroidia;D_3__Bacteroidales;D_4__Prevotellaceae;D_5__Prevotella 7;__
D_0__Bacteria;D_1__Firmicutes;D_2__Bacilli;D_3__Bacillales;D_4__Paenibacillaceae;D_5__Brevibacillus;__
D_0__Bacteria;D_1__Firmicutes;D_2__Bacilli;D_3__Lactobacillales;D_4__Streptococcaceae;D_5__Streptococcus;D_6__Streptococcus hyovaginalis
D_0__Bacteria;D_1__Patescibacteria;D_2__Parcubacteria;D_3__Candidatus Nomurabacteria;__;__;__
D_0__Bacteria;D_1__Firmicutes;D_2__Clostridia;D_3__Clostridiales;D_4__Lachnospiraceae;D_5__Coprococcus 3;__
D_0__Bacteria;D_1__Actinobacteria;D_2__Coriobacteriia;D_3__Coriobacteriales;D_4__Atopobiaceae;D_5__Libanicoccus;D_6__uncultured bacterium
D_0__Bacteria;D_1__Firmicutes;D_2__Clostridia;D_3__Clostridiales;D_4__Ruminococcaceae;D_5__Ruminococcaceae NK4A214 group;__
D_0__Bacteria;D_1__Bacteroidetes;D_2__Bacteroidia;D_3__Bacteroidales;D_4__Prevotellaceae;D_5__Prevotellaceae UCG-004;D_6__uncultured bacterium
D_0__Bacteria;D_1__Actinobacteria;D_2__Actinobacteria;D_3__Corynebacteriales;D_4__Corynebacteriaceae;D_5__Corynebacterium 1;D_6__Corynebacterium stationis
D_0__Archaea;D_1__Euryarchaeota;D_2__Thermoplasmata;D_3__Marine Group III;D_4__uncultured archaeon;D_5__uncultured archaeon;D_6__uncultured archaeon
D_0__Bacteria;D_1__Firmicutes;D_2__Clostridia;D_3__Clostridiales;D_4__Ruminococcaceae;D_5__Ruminococcus 2;__
D_0__Bacteria;D_1__Bacteroidetes;D_2__Bacteroidia;D_3__Bacteroidales;D_4__Porphyromonadaceae;D_5__Porphyromonas;__
D_0__Bacteria;D_1__Proteobacteria;D_2__Deltaproteobacteria;D_3__Desulfuromonadales;D_4__Geobacteraceae;D_5__Geobacter;__
D_0__Bacteria;D_1__Firmicutes;D_2__Bacilli;D_3__Lactobacillales;D_4__Aerococcaceae;D_5__uncultured;D_6__uncultured bacterium
D_0__Bacteria;D_1__Actinobacteria;D_2__Coriobacteriia;D_3__Coriobacteriales;D_4__Atopobiaceae;D_5__Olsenella;D_6__uncultured bacterium
D_0__Bacteria;D_1__Firmicutes;D_2__Erysipelotrichia;D_3__Erysipelotrichales;D_4__Erysipelotrichaceae;D_5__Solobacterium;__
D_0__Bacteria;D_1__Bacteroidetes;D_2__Bacteroidia;D_3__Bacteroidales;D_4__Bacteroidaceae;D_5__Bacteroides;D_6__Bacteroides neonati
D_0__Bacteria;D_1__Firmicutes;D_2__Erysipelotrichia;D_3__Erysipelotrichales;D_4__Erysipelotrichaceae;D_5__Erysipelothrix;D_6__uncultured bacterium
D_0__Bacteria;D_1__Bacteroidetes;D_2__Bacteroidia;D_3__Bacteroidales;D_4__Prevotellaceae;D_5__Alloprevotella;D_6__Prevotellamassilia timonensis
D_0__Bacteria;D_1__Proteobacteria;D_2__Deltaproteobacteria;D_3__Myxococcales;D_4__Blrii41;D_5__uncultured bacterium;D_6__uncultured bacterium
D_0__Bacteria;D_1__Firmicutes;D_2__Clostridia;D_3__Clostridiales;D_4__Ruminococcaceae;D_5__Ruminococcus 1;D_6__Ruminococcus sp. HUN007
D_0__Bacteria;D_1__Bacteroidetes;D_2__Bacteroidia;D_3__Cytophagales;D_4__Spirosomaceae;D_5__Dyadobacter;__
D_0__Bacteria;D_1__Actinobacteria;D_2__Actinobacteria;D_3__Corynebacteriales;D_4__Corynebacteriaceae;D_5__Corynebacterium 1;D_6__Corynebacterium xerosis
D_0__Bacteria;D_1__Actinobacteria;D_2__Actinobacteria;D_3__Corynebacteriales;D_4__Corynebacteriaceae;D_5__Corynebacterium 1;D_6__uncultured Corynebacterium sp.
D_0__Bacteria;D_1__Firmicutes;D_2__Erysipelotrichia;D_3__Erysipelotrichales;D_4__Erysipelotrichaceae;D_5__Holdemanella;__
D_0__Bacteria;D_1__Firmicutes;D_2__Bacilli;D_3__Bacillales;D_4__Bacillaceae;D_5__Bacillus;D_6__Streptococcus pneumoniae
D_0__Bacteria;D_1__Proteobacteria;D_2__Gammaproteobacteria;D_3__Aeromonadales;D_4__Aeromonadaceae;D_5__Aeromonas;__
D_0__Bacteria;D_1__Firmicutes;D_2__Clostridia;D_3__Clostridiales;D_4__Ruminococcaceae;D_5__Ruminococcaceae UCG-004;D_6__uncultured rumen bacterium
D_0__Bacteria;D_1__Tenericutes;D_2__Mollicutes;D_3__Mollicutes RF39;D_4__metagenome;D_5__metagenome;D_6__metagenome
D_0__Bacteria;D_1__Patescibacteria;D_2__Parcubacteria;D_3__Candidatus Zambryskibacteria;D_4__Parcubacteria group bacterium GW2011_GWA2_40_14;D_5__Parcubacteria group bacterium GW2011_GWA2_40_14;D_6__Parcuba
D_0__Bacteria;D_1__Firmicutes;D_2__Bacilli;D_3__Lactobacillales;D_4__Carnobacteriaceae;D_5__Desemzia;__
D_0__Bacteria;D_1__Firmicutes;D_2__Clostridia;D_3__Clostridiales;D_4__Family XIII;D_5__Family XIII AD3011 group;D_6__uncultured Eubacterium sp.
D_0__Bacteria;D_1__Bacteroidetes;D_2__Bacteroidia;D_3__Flavobacteriales;D_4__Weeksellaceae;D_5__Empedobacter;D_6__Empedobacter brevis
D_0__Bacteria;D_1__Firmicutes;D_2__Clostridia;D_3__Clostridiales;D_4__Ruminococcaceae;D_5__Ruminococcaceae UCG-014;D_6__uncultured Ruminococcaceae bacterium
D_0__Bacteria;D_1__Firmicutes;D_2__Clostridia;D_3__Clostridiales;D_4__Ruminococcaceae;D_5__Ruminococcaceae UCG-010;D_6__metagenome
D_0__Bacteria;D_1__Bacteroidetes;D_2__Bacteroidia;D_3__Bacteroidales;D_4__Prevotellaceae;D_5__Prevotella 7;D_6__uncultured rumen bacterium
D_0__Bacteria;D_1__Firmicutes;D_2__Negativicutes;D_3__Selenomonadales;D_4__Veillonellaceae;D_5__Dialister;D_6__uncultured bacterium
D_0__Bacteria;D_1__Firmicutes;D_2__Clostridia;D_3__Clostridiales;D_4__Lachnospiraceae;D_5__Lachnospira;__
D_0__Bacteria;D_1__Proteobacteria;D_2__Deltaproteobacteria;__;__;__;__
D_0__Bacteria;D_1__Bacteroidetes;D_2__Bacteroidia;D_3__Bacteroidales;D_4__Prevotellaceae;D_5__Prevotella 7;D_6__uncultured Prevotella sp.
D_0__Bacteria;D_1__Firmicutes;D_2__Clostridia;D_3__Clostridiales;D_4__Clostridiales vadinBB60 group;D_5__uncultured organism;D_6__uncultured organism
D_0__Bacteria;D_1__FCPU426;D_2__metagenome;D_3__metagenome;D_4__metagenome;D_5__metagenome;D_6__metagenome
D_0__Bacteria;D_1__Firmicutes;D_2__Clostridia;D_3__Clostridiales;D_4__Syntrophomonadaceae;D_5__Syntrophomonas;D_6__Syntrophomonas cellicola
D_0__Bacteria;D_1__Proteobacteria;D_2__Gammaproteobacteria;D_3__Salinisphaerales;D_4__Solimonadaceae;D_5__Polycyclovorans;D_6__uncultured bacterium
D_0__Bacteria;D_1__Firmicutes;D_2__Clostridia;D_3__Clostridiales;D_4__Family XIII;D_5__Mogibacterium;D_6__uncultured bacterium
D_0__Bacteria;D_1__Firmicutes;D_2__Erysipelotrichia;D_3__Erysipelotrichales;D_4__Erysipelotrichaceae;D_5__Erysipelothrix;D_6__uncultured organism
D_0__Bacteria;D_1__Firmicutes;D_2__Clostridia;D_3__Clostridiales;D_4__Ruminococcaceae;D_5__Ruminococcaceae UCG-002;D_6__uncultured organism
D_0__Bacteria;D_1__Bacteroidetes;D_2__Bacteroidia;D_3__Sphingobacteriales;D_4__AKYH767;D_5__uncultured bacterium;D_6__uncultured bacterium
D_0__Bacteria;D_1__Proteobacteria;D_2__Gammaproteobacteria;D_3__Betaproteobacteriales;D_4__Neisseriaceae;D_5__Neisseria;D_6__Neisseria dentiae

D_0__Bacteria;D_1__Proteobacteria;D_2__Gammaproteobacteria;D_3__Betaproteobacteriales;D_4__Neisseriaceae;D_5__Neisseria;D_6__Neisseria dentiae
D_0__Bacteria;D_1__Proteobacteria;D_2__Deltaproteobacteria;D_3__Myxococcales;D_4__Phaselicystidaceae;D_5__Phaselicystis;D_6__uncultured bacterium
D_0__Bacteria;D_1__Planctomycetes;D_2__Planctomycetacia;D_3__Gemmatales;D_4__Gemmataceae;D_5__uncultured;D_6__uncultured Planctomycetia bacterium
D_0__Bacteria;D_1__Lentisphaerae;D_2__Lentisphaeria;D_3__Victivallales;D_4__Victivallaceae;D_5__uncultured bacterium;D_6__uncultured bacterium
D_0__Bacteria;D_1__Firmicutes;D_2__Clostridia;D_3__Clostridiales;D_4__Lachnospiraceae;D_5__Dorea;__
D_0__Bacteria;D_1__Proteobacteria;D_2__Alphaproteobacteria;D_3__Rhizobiales;D_4__Beijerinckiaceae;D_5__Methylovirgula;D_6__uncultured bacterium
D_0__Bacteria;D_1__Firmicutes;D_2__Clostridia;D_3__Clostridiales;D_4__Ruminococcaceae;D_5__uncultured;D_6__gut metagenome
D_0__Bacteria;D_1__Firmicutes;D_2__Clostridia;D_3__Clostridiales;D_4__Ruminococcaceae;D_5__Ruminococcus 2;D_6__uncultured Ruminococcaceae bacterium
D_0__Bacteria;D_1__Proteobacteria;D_2__Alphaproteobacteria;D_3__Rhizobiales;D_4__Devosiaceae;D_5__Devosia;__
D_0__Bacteria;D_1__Actinobacteria;D_2__Actinobacteria;D_3__Propionibacteriales;D_4__Nocardioidaceae;D_5__Nocardioides;__
D_0__Bacteria;D_1__Firmicutes;D_2__Clostridia;D_3__Clostridiales;D_4__Lachnospiraceae;D_5__Roseburia;D_6__uncultured bacterium
D_0__Bacteria;D_1__Firmicutes;D_2__Negativicutes;D_3__Selenomonadales;D_4__Veillonellaceae;D_5__Mitsuokella;D_6__metagenome
D_0__Bacteria;D_1__Firmicutes;D_2__Clostridia;D_3__Clostridiales;D_4__Lachnospiraceae;D_5__Lachnospiraceae NK4A136 group;D_6__uncultured prokaryote
D_0__Bacteria;D_1__Firmicutes;D_2__Clostridia;D_3__Clostridiales;D_4__Lachnospiraceae;D_5__[Eubacterium] xylanophilum group;__
D_0__Bacteria;D_1__Actinobacteria;D_2__Coriobacteriia;D_3__Coriobacteriales;D_4__Atopobiaceae;D_5__uncultured;D_6__uncultured bacterium
D_0__Bacteria;D_1__Patescibacteria;D_2__Parcubacteria;D_3__Candidatus Yonathbacteria;D_4__uncultured bacterium;D_5__uncultured bacterium;D_6__uncultured bacterium
D_0__Bacteria;D_1__Proteobacteria;D_2__Gammaproteobacteria;D_3__Betaproteobacteriales;D_4__Burkholderiaceae;D_5__Castellaniella;__
D_0__Bacteria;D_1__Proteobacteria;D_2__Gammaproteobacteria;D_3__Beggiatoales;D_4__Beggiatoaceae;D_5__uncultured;D_6__uncultured bacterium
D_0__Bacteria;D_1__Cyanobacteria;D_2__Melainabacteria;D_3__Gastranaerophilales;__;__;__
D_0__Bacteria;D_1__Rokubacteria;D_2__NC10;D_3__Rokubacteriales;D_4__uncultured bacterium;D_5__uncultured bacterium;D_6__uncultured bacterium
D_0__Bacteria;D_1__Firmicutes;D_2__Bacilli;D_3__Bacillales;D_4__Bacillaceae;D_5__Bacillus;D_6__Bacillus thermoamylovorans
D_0__Bacteria;D_1__Proteobacteria;D_2__Gammaproteobacteria;D_3__Betaproteobacteriales;D_4__Rhodocyclaceae;D_5__Azoarcus;D_6__Azoarcus tolulyticus
D_0__Bacteria;D_1__Bacteroidetes;D_2__Bacteroidia;D_3__Bacteroidales;D_4__Prevotellaceae;D_5__Alloprevotella;D_6__uncultured Bacteroidales bacterium
D_0__Bacteria;D_1__Patescibacteria;D_2__Parcubacteria;D_3__Candidatus Yanofskybacteria;D_4__uncultured bacterium;D_5__uncultured bacterium;D_6__uncultured bacterium
D_0__Bacteria;D_1__Proteobacteria;D_2__Deltaproteobacteria;D_3__Myxococcales;D_4__Haliangiaceae;D_5__Haliangium;D_6__uncultured bacterium
D_0__Bacteria;D_1__Firmicutes;D_2__Clostridia;D_3__Clostridiales;D_4__Ruminococcaceae;D_5__CAG-352;D_6__uncultured organism
D_0__Bacteria;D_1__Firmicutes;D_2__Negativicutes;D_3__Selenomonadales;D_4__Veillonellaceae;D_5__Selenomonas;D_6__Selenomonas lacticifex
D_0__Bacteria;D_1__Bacteroidetes;D_2__Bacteroidia;D_3__Bacteroidales;D_4__Prevotellaceae;D_5__Prevotella 7;D_6__Prevotella scopos JCM 17725
D_0__Bacteria;D_1__Actinobacteria;D_2__Acidimicrobiia;D_3__Microtrichales;D_4__Iamiaceae;D_5__Iamia;D_6__uncultured bacterium
D_0__Bacteria;D_1__Firmicutes;D_2__Clostridia;D_3__Clostridiales;D_4__Ruminococcaceae;D_5__Ethanoligenens;D_6__uncultured bacterium
D_0__Bacteria;D_1__Proteobacteria;D_2__Gammaproteobacteria;D_3__Betaproteobacteriales;D_4__Burkholderiaceae;D_5__Curvibacter;D_6__Curvibacter gracilis
D_0__Bacteria;D_1__Firmicutes;D_2__Bacilli;D_3__Lactobacillales;D_4__Aerococcaceae;D_5__Aerococcus;D_6__Aerococcus suis
D_0__Bacteria;D_1__Bacteroidetes;D_2__Bacteroidia;D_3__Bacteroidales;D_4__Tannerellaceae;D_5__Parabacteroides;D_6__Porphyromonadaceae bacterium DJF_B175
D_0__Bacteria;D_1__Bacteroidetes;D_2__Bacteroidia;D_3__Bacteroidales;D_4__Prevotellaceae;D_5__Prevotellaceae NK3B31 group;D_6__metagenome
D_0__Bacteria;D_1__Actinobacteria;D_2__Actinobacteria;D_3__Frankiales;D_4__Cryptosporangiaceae;D_5__Fodinicola;D_6__metagenome
D_0__Bacteria;D_1__Proteobacteria;D_2__Gammaproteobacteria;D_3__Pseudomonadales;D_4__Moraxellaceae;D_5__Acinetobacter;D_6__Acinetobacter rudis CIP 110305
D_0__Bacteria;D_1__Firmicutes;D_2__Clostridia;D_3__Clostridiales;D_4__Lachnospiraceae;D_5__Lachnospiraceae FCS020 group;__
D_0__Bacteria;D_1__Actinobacteria;D_2__Actinobacteria;D_3__Corynebacteriales;D_4__Corynebacteriaceae;D_5__Corynebacterium;D_6__Corynebacterium pollutisoli
D_0__Bacteria;D_1__Proteobacteria;D_2__Gammaproteobacteria;D_3__Betaproteobacteriales;D_4__Burkholderiaceae;D_5__Duganella;D_6__uncultured bacterium
D_0__Bacteria;D_1__Proteobacteria;__;__;__;__;__
D_0__Bacteria;D_1__Actinobacteria;D_2__Coriobacteriia;D_3__Coriobacteriales;D_4__Eggerthellaceae;D_5__DNF00809;D_6__uncultured bacterium
D_0__Bacteria;D_1__Proteobacteria;D_2__Alphaproteobacteria;D_3__Caulobacterales;D_4__Caulobacteraceae;D_5__Phenylobacterium;__
D_0__Bacteria;D_1__Cyanobacteria;D_2__Oxyphotobacteria;D_3__Chloroplast;D_4__Vischeria sp. CAUP Q 202;D_5__Vischeria sp. CAUP Q 202;D_6__Vischeria sp. CAUP Q 202
D_0__Bacteria;D_1__Chlamydiae;D_2__Chlamydiae;D_3__Chlamydiales;__;__;__
D_0__Bacteria;D_1__Actinobacteria;D_2__Actinobacteria;D_3__Micrococcales;D_4__Micrococcaceae;D_5__Kocuria;__
D_0__Bacteria;D_1__Actinobacteria;D_2__Actinobacteria;D_3__Bifidobacteriales;D_4__Bifidobacteriaceae;D_5__Bifidobacterium;D_6__Bifidobacterium minimum
D_0__Bacteria;D_1__Firmicutes;D_2__Clostridia;D_3__Clostridiales;D_4__Lachnospiraceae;D_5__[Eubacterium] hallii group;D_6__uncultured bacterium
D_0__Bacteria;D_1__Actinobacteria;D_2__Actinobacteria;D_3__Corynebacteriales;D_4__Corynebacteriaceae;D_5__Corynebacterium 1;D_6__Corynebacterium jeikeium
D_0__Bacteria;D_1__Actinobacteria;D_2__Actinobacteria;D_3__Actinomycetales;D_4__Actinomycetaceae;D_5__Flaviflexus;D_6__uncultured bacterium
D_0__Bacteria;D_1__Proteobacteria;D_2__Gammaproteobacteria;D_3__Betaproteobacteriales;D_4__Burkholderiaceae;D_5__Pelomonas;__
D_0__Bacteria;D_1__Firmicutes;D_2__Negativicutes;D_3__Selenomonadales;D_4__Veillonellaceae;D_5__uncultured;D_6__uncultured rumen bacterium 3C28d-3
D_0__Bacteria;D_1__Firmicutes;D_2__Clostridia;D_3__Clostridiales;D_4__Clostridiaceae 1;D_5__Clostridium sensu stricto 12;D_6__Clostridium acidisoli
D_0__Bacteria;D_1__Bacteroidetes;D_2__Bacteroidia;D_3__Bacteroidales;D_4__Prevotellaceae;D_5__Prevotella 1;D_6__uncultured bacterium
D_0__Bacteria;D_1__Proteobacteria;D_2__Alphaproteobacteria;D_3__Rickettsiales;D_4__Rickettsiaceae;__;__
D_0__Bacteria;D_1__Chloroflexi;D_2__JG30-KF-CM66;D_3__metagenome;D_4__metagenome;D_5__metagenome;D_6__metagenome
D_0__Archaea;D_1__Diapherotrites;D_2__Micrarchaeia;D_3__uncultured euryarchaeote;D_4__uncultured euryarchaeote;D_5__uncultured euryarchaeote;D_6__uncultured euryarchaeote
D_0__Bacteria;D_1__Firmicutes;D_2__Erysipelotrichia;D_3__Erysipelotrichales;D_4__Erysipelotrichaceae;D_5__Sharpea;D_6__uncultured bacterium
D_0__Bacteria;D_1__Patescibacteria;D_2__Parcubacteria;D_3__Candidatus Nomurabacteria;D_4__uncultured bacterium;D_5__uncultured bacterium;D_6__uncultured bacterium
D_0__Bacteria;D_1__Proteobacteria;D_2__Gammaproteobacteria;D_3__Cardiobacteriales;D_4__Wohlfahrtiimonadaceae;D_5__Ignatzschineria;__
D_0__Bacteria;D_1__Proteobacteria;D_2__Gammaproteobacteria;D_3__Betaproteobacteriales;D_4__Rhodocyclaceae;D_5__Denitratisoma;D_6__uncultured bacterium
D_0__Bacteria;D_1__Omnitrophicaeota;D_2__uncultured bacterium;D_3__uncultured bacterium;D_4__uncultured bacterium;D_5__uncultured bacterium;D_6__uncultured bacterium
D_0__Bacteria;D_1__Firmicutes;D_2__Clostridia;D_3__Clostridiales;D_4__Ruminococcaceae;D_5__Ruminococcaceae NK4A214 group;D_6__uncultured rumen bacterium
D_0__Bacteria;D_1__Firmicutes;D_2__Negativicutes;D_3__Selenomonadales;D_4__Veillonellaceae;D_5__Megasphaera;D_6__Megasphaera sp. MH
D_0__Bacteria;D_1__Proteobacteria;D_2__Deltaproteobacteria;D_3__Myxococcales;D_4__Vulgatibacteraceae;D_5__Vulgatibacter;D_6__Vulgatibacter incomptus
D_0__Bacteria;D_1__Patescibacteria;D_2__Parcubacteria;D_3__Candidatus Kaiserbacteria;D_4__groundwater metagenome;D_5__groundwater metagenome;D_6__groundwater metagenome
D_0__Bacteria;D_1__Bacteroidetes;D_2__Bacteroidia;D_3__Sphingobacteriales;D_4__Sphingobacteriaceae;D_5__Mucilaginibacter;D_6__uncultured bacterium
D_0__Bacteria;D_1__Actinobacteria;D_2__Actinobacteria;D_3__Corynebacteriales;D_4__Corynebacteriaceae;D_5__Corynebacterium;__
D_0__Bacteria;D_1__Tenericutes;D_2__Mollicutes;D_3__Mycoplasmatales;D_4__Mycoplasmataceae;D_5__Mycoplasma;__
D_0__Bacteria;D_1__Chloroflexi;D_2__Anaerolineae;D_3__SBR1031;D_4__A4b;D_5__uncultured bacterium;D_6__uncultured bacterium
D_0__Bacteria;D_1__Firmicutes;D_2__Bacilli;D_3__Bacillales;D_4__Paenibacillaceae;D_5__Paenibacillus;__
D_0__Bacteria;D_1__Bacteroidetes;D_2__Bacteroidia;D_3__Flavobacteriales;D_4__Flavobacteriaceae;D_5__Flavobacterium;D_6__Flavobacterium sp. IGB 4-14
D_0__Bacteria;D_1__Patescibacteria;D_2__Microgenomatia;D_3__Candidatus Pacebacteria;D_4__uncultured soil bacterium;D_5__uncultured soil bacterium;D_6__uncultured soil bacterium
D_0__Bacteria;D_1__Actinobacteria;D_2__Actinobacteria;D_3__Streptomycetales;D_4__Streptomycetaceae;D_5__Streptomyces;__
D_0__Bacteria;D_1__Patescibacteria;D_2__Parcubacteria;D_3__Candidatus Nomurabacteria;D_4__Candidatus Nomurabacteria bacterium RIFCSPLOWO2_01_FULL_42_20;D_5__Candidatus Nomurabacteria bacterium RIFCSPLOWO2
D_0__Bacteria;D_1__Patescibacteria;D_2__Parcubacteria;D_3__Candidatus Colwellbacteria;D_4__uncultured bacterium;D_5__uncultured bacterium;D_6__uncultured bacterium
D_0__Bacteria;D_1__Proteobacteria;D_2__Alphaproteobacteria;D_3__Rickettsiales;D_4__Mitochondria;D_5__uncultured bacterium;D_6__uncultured bacterium
D_0__Bacteria;D_1__Firmicutes;D_2__Erysipelotrichia;D_3__Erysipelotrichales;D_4__Erysipelotrichaceae;D_5__[Anaerorhabdus] furcosa group;D_6__uncultured bacterium
D_0__Bacteria;D_1__WPS-2;D_2__metagenome;D_3__metagenome;D_4__metagenome;D_5__metagenome;D_6__metagenome
D_0__Bacteria;D_1__Firmicutes;D_2__Clostridia;D_3__Clostridiales;D_4__Peptococcaceae;D_5__Desulfosporosinus;__

D_0__Bacteria;D_1__Proteobacteria;D_2__Gammaproteobacteria;D_3__Betaproteobacteriales;D_4__Burkholderiaceae;D_5__Janthinobacterium;__

D_0__Bacteria;D_1__Actinobacteria;D_2__Actinobacteria;D_3__Micrococcales;D_4__Microbacteriaceae;D_5__Microbacterium;__

D_0__Bacteria;D_1__Actinobacteria;D_2__Actinobacteria;D_3__Micrococcales;D_4__Micrococcaceae;D_5__Rothia;__

D_0__Bacteria;D_1__Firmicutes;D_2__Clostridia;D_3__Clostridiales;D_4__Ruminococcaceae;D_5__Ruminococcaceae UCG-005;D_6__uncultured rumen bacterium

D_0__Bacteria;D_1__Bacteroidetes;D_2__Bacteroidia;D_3__Sphingobacteriales;D_4__Sphingobacteriaceae;D_5__Sphingobacterium;__

D_0__Bacteria;D_1__Firmicutes;D_2__Negativicutes;D_3__Selenomonadales;D_4__Acidaminococcaceae;D_5__Acidaminococcus;D_6__Acidaminococcus massiliensis

D_0__Bacteria;D_1__Fusobacteria;D_2__Fusobacteriia;D_3__Fusobacteriales;D_4__Fusobacteriaceae;D_5__Fusobacterium;D_6__Fusobacterium necrophorum subsp. necrophorum

D_0__Bacteria;D_1__Actinobacteria;D_2__Coriobacteriia;D_3__Coriobacteriales;D_4__Coriobacteriaceae;D_5__Collinsella;__

D_0__Bacteria;D_1__Proteobacteria;D_2__Alphaproteobacteria;D_3__Caulobacterales;D_4__Caulobacteraceae;D_5__Caulobacter;__

D_0__Bacteria;D_1__Firmicutes;D_2__Clostridia;D_3__Clostridiales;D_4__Christensenellaceae;D_5__Christensenellaceae R-7 group;D_6__uncultured bacterium

D_0__Bacteria;D_1__Firmicutes;D_2__Bacilli;D_3__Bacillales;D_4__Staphylococcaceae;D_5__Staphylococcus;D_6__Staphylococcus chromogenes

D_0__Bacteria;D_1__Tenericutes;D_2__Mollicutes;D_3__Mollicutes RF39;D_4__uncultured bacterium;D_5__uncultured bacterium;D_6__uncultured bacterium

D_0__Bacteria;D_1__Planctomycetes;D_2__Planctomycetacia;D_3__Gemmatales;D_4__Gemmataceae;D_5__Gemmata;D_6__uncultured bacterium

D_0__Bacteria;D_1__Chlamydiae;D_2__Chlamydiae;D_3__Chlamydiales;D_4__Simkaniaceae;D_5__uncultured;D_6__uncultured Candidatus Rhabdochlamydia sp.

D_0__Bacteria;D_1__Actinobacteria;D_2__Actinobacteria;D_3__Corynebacteriales;D_4__Mycobacteriaceae;D_5__Mycobacterium;__

D_0__Bacteria;D_1__Proteobacteria;D_2__Gammaproteobacteria;D_3__Pasteurellales;D_4__Pasteurellaceae;D_5__Pasteurella;D_6__Pasteurella multocida

D_0__Bacteria;D_1__Bacteroidetes;D_2__Bacteroidia;D_3__Bacteroidales;D_4__Rikenellaceae;D_5__Rikenellaceae RC9 gut group;D_6__gut metagenome

D_0__Bacteria;D_1__Firmicutes;D_2__Clostridia;D_3__Clostridiales;D_4__Lachnospiraceae;D_5__Coprococcus 1;__

D_0__Bacteria;D_1__Actinobacteria;D_2__Coriobacteriia;D_3__Coriobacteriales;D_4__Eggerthellaceae;D_5__Slackia;__

D_0__Bacteria;D_1__Firmicutes;D_2__Clostridia;D_3__Clostridiales;D_4__Lachnospiraceae;D_5__Lachnoclostridium;__

D_0__Bacteria;D_1__Firmicutes;D_2__Clostridia;D_3__Clostridiales;D_4__Family XIII;D_5__Family XIII UCG-001;D_6__uncultured Anaerovorax sp.

D_0__Bacteria;D_1__Actinobacteria;D_2__Actinobacteria;D_3__Corynebacteriales;D_4__Corynebacteriaceae;D_5__Corynebacterium 1;D_6__Corynebacterium suicordis

D_0__Bacteria;D_1__Proteobacteria;D_2__Gammaproteobacteria;__;__;__;__

D_0__Bacteria;D_1__Proteobacteria;D_2__Deltaproteobacteria;D_3__Bdellovibrionales;D_4__Bdellovibrionaceae;D_5__Bdellovibrio;D_6__uncultured bacterium

D_0__Bacteria;D_1__Firmicutes;D_2__Clostridia;D_3__Clostridiales;D_4__Peptostreptococcaceae;D_5__Peptostreptococcus;__

D_0__Bacteria;D_1__Firmicutes;D_2__Bacilli;D_3__Lactobacillales;D_4__Carnobacteriaceae;D_5__Carnobacterium;__

D_0__Bacteria;D_1__Bacteroidetes;D_2__Bacteroidia;D_3__Chitinophagales;D_4__Chitinophagaceae;D_5__Filobacterium;D_6__cilia-associated respiratory bacterium 95-15405

D_0__Bacteria;D_1__Patescibacteria;D_2__Parcubacteria;D_3__Candidatus Vogelbacteria;D_4__uncultured bacterium;D_5__uncultured bacterium;D_6__uncultured bacterium

D_0__Bacteria;D_1__Proteobacteria;D_2__Gammaproteobacteria;D_3__Oceanospirillales;D_4__Halomonadaceae;D_5__Halomonas;__

D_0__Bacteria;D_1__Firmicutes;D_2__Clostridia;D_3__Clostridiales;D_4__Ruminococcaceae;D_5__Ruminococcaceae UCG-009;D_6__uncultured bacterium

D_0__Bacteria;D_1__Actinobacteria;D_2__Coriobacteriia;D_3__Coriobacteriales;D_4__Eggerthellaceae;D_5__uncultured;D_6__uncultured bacterium

D_0__Bacteria;D_1__Actinobacteria;D_2__Coriobacteriia;D_3__Coriobacteriales;D_4__Atopobiaceae;D_5__metagenome;D_6__metagenome

D_0__Bacteria;D_1__Proteobacteria;D_2__Alphaproteobacteria;D_3__Caulobacterales;D_4__Caulobacteraceae;D_5__uncultured;D_6__uncultured bacterium

D_0__Bacteria;D_1__Firmicutes;D_2__Clostridia;D_3__Clostridiales;D_4__Lachnospiraceae;D_5__Lachnospiraceae NK3A20 group;D_6__uncultured bacterium

D_0__Bacteria;D_1__Actinobacteria;D_2__Actinobacteria;D_3__Pseudonocardiales;D_4__Pseudonocardiaceae;D_5__Actinomycetospora;D_6__uncultured bacterium

D_0__Bacteria;D_1__Actinobacteria;D_2__Actinobacteria;D_3__Corynebacteriales;D_4__Corynebacteriaceae;D_5__Corynebacterium 1;D_6__Corynebacterium pseudodiphtheriticum

D_0__Bacteria;D_1__Firmicutes;D_2__Clostridia;D_3__Clostridiales;D_4__Lachnospiraceae;D_5__Lachnospiraceae NK3A20 group;D_6__uncultured rumen bacterium 4C3d-2

D_0__Bacteria;D_1__Bacteroidetes;D_2__Bacteroidia;D_3__Bacteroidales;D_4__Porphyromonadaceae;D_5__Porphyromonas;D_6__uncultured bacterium

D_0__Bacteria;D_1__Proteobacteria;D_2__Alphaproteobacteria;D_3__Rhizobiales;D_4__Hyphomicrobiaceae;D_5__Hyphomicrobium;D_6__uncultured Hyphomicrobiaceae bacterium

D_0__Bacteria;D_1__Planctomycetes;D_2__OM190;D_3__uncultured bacterium;D_4__uncultured bacterium;D_5__uncultured bacterium;D_6__uncultured bacterium

D_0__Bacteria;D_1__Firmicutes;D_2__Clostridia;D_3__Clostridiales;D_4__Peptococcaceae;D_5__Peptococcus;D_6__uncultured bacterium

D_0__Bacteria;D_1__Actinobacteria;D_2__Acidimicrobiia;D_3__Microtrichales;D_4__Ilumatobacteraceae;D_5__Ilumatobacter;D_6__metagenome

D_0__Bacteria;D_1__Firmicutes;D_2__Clostridia;D_3__Clostridiales;D_4__Lachnospiraceae;D_5__Lachnospiraceae NK3A20 group;__

D_0__Bacteria;D_1__Actinobacteria;D_2__Actinobacteria;D_3__Micrococcales;D_4__Micrococcaceae;D_5__Rothia;D_6__Rothia aerolata

D_0__Bacteria;D_1__Firmicutes;D_2__Clostridia;D_3__Clostridiales;D_4__Lachnospiraceae;D_5__Lachnospiraceae UCG-001;D_6__metagenome

D_0__Bacteria;D_1__Patescibacteria;D_2__Parcubacteria;D_3__Candidatus Jorgensenbacteria;__;__;__

D_0__Bacteria;D_1__Actinobacteria;D_2__Actinobacteria;D_3__Pseudonocardiales;D_4__Pseudonocardiaceae;D_5__Amycolatopsis;__

D_0__Bacteria;D_1__Omnitrophicaeota;D_2__uncultured beta proteobacterium;D_3__uncultured beta proteobacterium;D_4__uncultured beta proteobacterium;D_5__uncultured beta proteobacterium;D_6__uncultured beta proteobacteriu

D_0__Bacteria;D_1__Firmicutes;D_2__Clostridia;D_3__Clostridiales;D_4__Ruminococcaceae;D_5__Candidatus Soleaferrea;D_6__uncultured bacterium

D_0__Bacteria;D_1__Firmicutes;D_2__Clostridia;D_3__Clostridiales;D_4__Family XI;D_5__Parvimonas;__

D_0__Bacteria;D_1__Patescibacteria;D_2__Parcubacteria;D_3__Candidatus Azambacteria;__;__;__

D_0__Bacteria;D_1__Firmicutes;D_2__Clostridia;D_3__Clostridiales;D_4__Clostridiaceae 1;D_5__Clostridium sensu stricto 12;__

D_0__Bacteria;D_1__Patescibacteria;D_2__Microgenomatia;D_3__Candidatus Pacebacteria;D_4__uncultured bacterium;D_5__uncultured bacterium;D_6__uncultured bacterium

D_0__Bacteria;D_1__Firmicutes;D_2__Clostridia;D_3__Clostridiales;D_4__Ruminococcaceae;D_5__Ruminococcaceae UCG-002;D_6__uncultured rumen bacterium

D_0__Bacteria;D_1__Actinobacteria;D_2__Actinobacteria;D_3__Propionibacteriales;D_4__Propionibacteriaceae;D_5__Propionimicrobium;D_6__Propionimicrobium lymphophilum DSM 4903

D_0__Bacteria;D_1__Firmicutes;D_2__Clostridia;D_3__Clostridiales;D_4__Ruminococcaceae;D_5__Ruminococcaceae UCG-010;D_6__uncultured bacterium

D_0__Bacteria;D_1__Patescibacteria;D_2__WS6 (Dojkabacteria);D_3__uncultured bacterium;D_4__uncultured bacterium;D_5__uncultured bacterium;D_6__uncultured bacterium

D_0__Bacteria;D_1__Firmicutes;D_2__Bacilli;D_3__Lactobacillales;D_4__Aerococcaceae;D_5__Globicatella;D_6__Globicatella sp. canine oral taxon 218

D_0__Bacteria;D_1__Actinobacteria;D_2__Actinobacteria;D_3__Propionibacteriales;D_4__Propionibacteriaceae;D_5__Tessaracoccus;D_6__uncultured bacterium

D_0__Bacteria;D_1__Patescibacteria;D_2__Parcubacteria;D_3__Candidatus Kaiserbacteria;__;__;__

D_0__Bacteria;D_1__Firmicutes;D_2__Clostridia;D_3__Clostridiales;D_4__Ruminococcaceae;__;__

D_0__Bacteria;D_1__Proteobacteria;D_2__Gammaproteobacteria;D_3__Betaproteobacteriales;D_4__Burkholderiaceae;D_5__Massilia;D_6__Massilia alkalitolerans

D_0__Bacteria;D_1__Proteobacteria;D_2__Gammaproteobacteria;D_3__Betaproteobacteriales;D_4__Burkholderiaceae;D_5__Castellaniella;D_6__Castellaniella defragrans

D_0__Bacteria;D_1__Firmicutes;D_2__Bacilli;D_3__Bacillales;D_4__Bacillaceae;D_5__Anaerobacillus;D_6__uncultured bacterium

D_0__Bacteria;D_1__Bacteroidetes;D_2__Bacteroidia;D_3__Flavobacteriales;D_4__Flavobacteriaceae;D_5__Flavobacterium;D_6__uncultured bacterium

D_0__Bacteria;D_1__Actinobacteria;D_2__Acidimicrobiia;D_3__Microtrichales;D_4__Ilumatobacteraceae;D_5__CL500-29 marine group;D_6__metagenome

D_0__Bacteria;D_1__Acidobacteria;D_2__Blastocatellia (Subgroup 4);D_3__11-24;D_4__uncultured bacterium;D_5__uncultured bacterium;D_6__uncultured bacterium

D_0__Bacteria;D_1__Proteobacteria;D_2__Alphaproteobacteria;D_3__Azospirillales;D_4__Azospirillaceae;D_5__Skermanella;__

D_0__Bacteria;D_1__Firmicutes;D_2__Negativicutes;D_3__Selenomonadales;D_4__Veillonellaceae;D_5__Pectinatus;__

D_0__Bacteria;D_1__Firmicutes;D_2__Clostridia;D_3__Clostridiales;D_4__Clostridiaceae 1;D_5__Clostridium sensu stricto 13;__

D_0__Bacteria;D_1__Firmicutes;D_2__Clostridia;D_3__Clostridiales;D_4__Clostridiaceae 1;D_5__Clostridium sensu stricto 11;__

D_0__Bacteria;D_1__Actinobacteria;D_2__Actinobacteria;D_3__Actinomycetales;D_4__Actinomycetaceae;D_5__Actinotignum;D_6__uncultured bacterium

D_0__Bacteria;D_1__Chloroflexi;D_2__Anaerolineae;D_3__Anaerolineales;D_4__Anaerolineaceae;D_5__uncultured;D_6__bacterium enrichment culture clone BA53

D_0__Bacteria;D_1__Proteobacteria;D_2__Alphaproteobacteria;D_3__Acetobacterales;D_4__Acetobacteraceae;D_5__Roseomonas;D_6__Roseomonas arctica

D_0__Bacteria;D_1__Chlamydiae;D_2__Chlamydiae;D_3__Chlamydiales;D_4__Chlamydiaceae;D_5__Chlamydia;D_6__Chlamydia suis

D_0__Bacteria;D_1__Bacteroidetes;D_2__Bacteroidia;D_3__Bacteroidales;D_4__Dysgonomonadaceae;D_5__Dysgonomonas;__

D_0__Bacteria;D_1__Patescibacteria;D_2__Microgenomatia;D_3__Candidatus Woesebacteria;__;__;__

D_0__Bacteria;D_1__Actinobacteria;D_2__Actinobacteria;__;__;__;__

D_0__Bacteria;D_1__Proteobacteria;D_2__Gammaproteobacteria;D_3__Enterobacteriales;D_4__Enterobacteriaceae;D_5__Raoultella;

D_0__Bacteria;D_1__Proteobacteria;D_2__Gammaproteobacteria;D_3__Enterobacteriales;D_4__Enterobacteriaceae;D_5__Raoultella;__

D_0__Bacteria;D_1__Proteobacteria;D_2__Gammaproteobacteria;D_3__Betaproteobacteriales;D_4__Burkholderiaceae;D_5__Diaphorobacter;D_6__uncultured compost bacterium

D_0__Bacteria;D_1__Firmicutes;D_2__Negativicutes;D_3__Selenomonadales;D_4__Veillonellaceae;D_5__Megasphaera;D_6__Megasphaera paucivorans

D_0__Bacteria;D_1__Actinobacteria;D_2__Thermoleophilia;D_3__Solirubrobacterales;D_4__67-14;D_5__metagenome;D_6__metagenome

D_0__Bacteria;D_1__Proteobacteria;D_2__Alphaproteobacteria;D_3__Acetobacterales;D_4__Acetobacteraceae;D_5__Acetobacter;__

D_0__Bacteria;D_1__Firmicutes;D_2__Clostridia;D_3__Clostridiales;D_4__Family XI;D_5__Murdochiella;D_6__uncultured bacterium

D_0__Bacteria;D_1__Bacteroidetes;D_2__Bacteroidia;D_3__Flavobacteriales;D_4__Flavobacteriaceae;D_5__Myroides;D_6__Myroides odoratimimus

D_0__Bacteria;D_1__Firmicutes;D_2__Bacilli;D_3__Lactobacillales;D_4__Enterococcaceae;D_5__Enterococcus;D_6__Enterococcus italicus

D_0__Bacteria;D_1__Cyanobacteria;D_2__Oxyphotobacteria;D_3__Chloroplast;D_4__Melosira varians;D_5__Melosira varians;D_6__Melosira varians

D_0__Bacteria;D_1__Firmicutes;D_2__Clostridia;D_3__Clostridiales;D_4__Ruminococcaceae;D_5__Ruminococcaceae UCG-014;D_6__gut metagenome

D_0__Archaea;D_1__Nanoarchaeaeota;D_2__Nanohaloarchaeia;D_3__Deep Sea Euryarchaeotic Group(DSEG);D_4__uncultured archaeon;D_5__uncultured archaeon;D_6__uncultured archaeon

D_0__Bacteria;D_1__Synergistetes;D_2__Synergistia;D_3__Synergistales;D_4__Synergistaceae;D_5__uncultured;D_6__bacterium enrichment culture clone BA27

D_0__Bacteria;D_1__Firmicutes;D_2__Erysipelotrichia;D_3__Erysipelotrichales;D_4__Erysipelotrichaceae;D_5__Catenisphaera;D_6__uncultured bacterium

D_0__Bacteria;D_1__Firmicutes;D_2__Bacilli;D_3__Lactobacillales;D_4__Lactobacillaceae;D_5__Lactobacillus;D_6__Lactobacillus harbinensis

D_0__Bacteria;D_1__Bacteroidetes;D_2__Bacteroidia;D_3__Bacteroidales;D_4__Muribaculaceae;D_5__metagenome;D_6__metagenome

D_0__Bacteria;D_1__Bacteroidetes;D_2__Bacteroidia;D_3__Bacteroidales;D_4__Bacteroidetes vadinHA17;D_5__metagenome;D_6__metagenome

D_0__Bacteria;D_1__Firmicutes;D_2__Clostridia;D_3__Clostridiales;D_4__Ruminococcaceae;D_5__Caproiciproducens;__

D_0__Bacteria;D_1__Bacteroidetes;D_2__Bacteroidia;D_3__Flavobacteriales;D_4__Weeksellaceae;D_5__Cloacibacterium;__

D_0__Bacteria;D_1__Proteobacteria;D_2__Gammaproteobacteria;D_3__Betaproteobacteriales;D_4__Burkholderiaceae;D_5__Comamonas;D_6__uncultured beta proteobacterium

D_0__Bacteria;D_1__Nitrospirae;D_2__Thermodesulfovibrionia;D_3__uncultured;D_4__uncultured bacterium;D_5__uncultured bacterium;D_6__uncultured bacterium

D_0__Bacteria;D_1__Gemmatimonadetes;D_2__Gemmatimonadetes;D_3__Gemmatimonadales;D_4__Gemmatimonadaceae;D_5__uncultured;D_6__denitrifying bacterium enrichment culture clone NOB_2_F8

D_0__Bacteria;D_1__Firmicutes;D_2__Clostridia;D_3__Clostridiales;D_4__Peptostreptococcaceae;D_5__Clostridioides;D_6__Clostridioides mangenotii

D_0__Bacteria;D_1__Firmicutes;D_2__Clostridia;D_3__Clostridiales;D_4__Clostridiaceae 1;D_5__Clostridium sensu stricto 11;D_6__uncultured bacterium

D_0__Bacteria;D_1__Firmicutes;D_2__Bacilli;D_3__Bacillales;D_4__Paenibacillaceae;D_5__Paenibacillus;D_6__Paenibacillus barengoltzii

D_0__Bacteria;D_1__Chlamydiae;D_2__Chlamydiae;D_3__Chlamydiales;D_4__Parachlamydiaceae;__;__

D_0__Bacteria;D_1__Bacteroidetes;D_2__Bacteroidia;D_3__Bacteroidales;D_4__Dysgonomonadaceae;D_5__Dysgonomonas;D_6__uncultured bacterium

D_0__Bacteria;D_1__Bacteroidetes;D_2__Bacteroidia;D_3__Bacteroidales;D_4__Bacteroidaceae;D_5__Bacteroides;__

D_0__Bacteria;D_1__Actinobacteria;D_2__Actinobacteria;D_3__Micrococcales;D_4__Micrococcaceae;D_5__Rothia;D_6__uncultured organism

D_0__Bacteria;D_1__Proteobacteria;D_2__Gammaproteobacteria;D_3__Betaproteobacteriales;D_4__Burkholderiaceae;D_5__Comamonas;__

D_0__Bacteria;D_1__Epsilonbacteraeota;D_2__Campylobacteria;D_3__Campylobacterales;D_4__Campylobacteraceae;D_5__Campylobacter;D_6__Campylobacter hyointestinalis subsp. lawsonii

D_0__Archaea;D_1__Euryarchaeota;D_2__Methanomicrobia;D_3__Methanosarcinales;D_4__Methanosaetaceae;D_5__Methanosaeta;D_6__uncultured archaeon

D_0__Bacteria;D_1__Bacteroidetes;D_2__Bacteroidia;D_3__Flavobacteriales;D_4__Weeksellaceae;D_5__Chryseobacterium;D_6__uncultured Flavobacteriaceae bacterium

D_0__Bacteria;D_1__Actinobacteria;D_2__Coriobacteriia;D_3__Coriobacteriales;D_4__Eggerthellaceae;D_5__Enterorhabdus;D_6__uncultured bacterium

D_0__Bacteria;D_1__Proteobacteria;D_2__Gammaproteobacteria;D_3__Pasteurellales;D_4__Pasteurellaceae;D_5__Haemophilus;__

D_0__Bacteria;D_1__Proteobacteria;D_2__Gammaproteobacteria;D_3__Betaproteobacteriales;D_4__Burkholderiaceae;D_5__Polaromonas;__

D_0__Bacteria;D_1__Proteobacteria;D_2__Alphaproteobacteria;D_3__Rhizobiales;D_4__Rhizobiales Incertae Sedis;D_5__Phreatobacter;D_6__uncultured Brucella sp.

D_0__Bacteria;D_1__Firmicutes;D_2__Bacilli;D_3__Lactobacillales;D_4__Streptococcaceae;D_5__Streptococcus;D_6__Streptococcus salivarius subsp. thermophilus

D_0__Bacteria;D_1__Thermotogae;D_2__Thermotogae;D_3__Petrotogales;D_4__Petrotogaceae;D_5__Defluviitoga;D_6__uncultured bacterium

D_0__Bacteria;D_1__Proteobacteria;D_2__Gammaproteobacteria;D_3__Betaproteobacteriales;D_4__Burkholderiaceae;D_5__Variovorax;__

D_0__Bacteria;D_1__Proteobacteria;D_2__Deltaproteobacteria;D_3__Syntrophobacterales;D_4__Syntrophobacteraceae;D_5__Syntrophobacter;__

D_0__Bacteria;D_1__Patescibacteria;D_2__Parcubacteria;D_3__Candidatus Adlerbacteria;D_4__metagenome;D_5__metagenome;D_6__metagenome

D_0__Bacteria;D_1__Firmicutes;D_2__Clostridia;D_3__Clostridiales;D_4__Ruminococcaceae;D_5__Ruminiclostridium;D_6__Clostridium merdae

D_0__Bacteria;D_1__Firmicutes;D_2__Bacilli;D_3__Lactobacillales;D_4__Carnobacteriaceae;D_5__Alloiococcus;D_6__uncultured bacterium

D_0__Bacteria;D_1__Bacteroidetes;D_2__Bacteroidia;D_3__Flavobacteriales;D_4__Weeksellaceae;D_5__Bergeyella;D_6__Cloacibacterium sp. canine oral taxon 320

D_0__Bacteria;D_1__Bacteroidetes;D_2__Bacteroidia;D_3__Bacteroidales;D_4__uncultured;D_5__uncultured bacterium;D_6__uncultured bacterium

D_0__Archaea;D_1__Diapherotrites;D_2__Iainarchaeia;D_3__Iainarchaeales;D_4__uncultured archaeon;D_5__uncultured archaeon;D_6__uncultured archaeon

D_0__Bacteria;D_1__Proteobacteria;D_2__Gammaproteobacteria;D_3__Coxiellales;D_4__Coxiellaceae;D_5__Coxiella;D_6__uncultured bacterium

D_0__Bacteria;D_1__Firmicutes;D_2__Clostridia;D_3__Clostridiales;D_4__Lachnospiraceae;D_5__Lachnospiraceae FCS020 group;D_6__metagenome

D_0__Bacteria;D_1__Firmicutes;D_2__Clostridia;D_3__Clostridiales;D_4__Lachnospiraceae;D_5__Lachnoclostridium 5;__

D_0__Bacteria;D_1__Bacteroidetes;D_2__Bacteroidia;D_3__Chitinophagales;D_4__Chitinophagaceae;D_5__Sediminibacterium;D_6__uncultured soil bacterium

D_0__Bacteria;D_1__Proteobacteria;D_2__Gammaproteobacteria;D_3__Acidiferrobacterales;D_4__Acidiferrobacteraceae;D_5__Sulfurifustis;D_6__uncultured bacterium

D_0__Bacteria;D_1__Bacteroidetes;D_2__Ignavibacteria;D_3__Ignavibacteriales;D_4__PHOS-HE36;D_5__uncultured bacterium;D_6__uncultured bacterium

D_0__Archaea;D_1__Euryarchaeota;D_2__Methanomicrobia;D_3__Methanomicrobiales;D_4__Methanoregulaceae;D_5__Methanoregula;D_6__uncultured euryarchaeote

D_0__Bacteria;D_1__Patescibacteria;D_2__Parcubacteria;D_3__Candidatus Nomurabacteria;D_4__Candidatus Nomurabacteria bacterium RIFCSPHIGHO2_02_FULL_38_15;D_5__Candidatus Nomurabacteria bacterium RIFCSPHIGHC
