# Supplementary figures and images for: Experimental Infection with Mycoplasma hyopneumoniae Strain 232 in Swine Influences the Lower Respiratory Microbiota
Source: Vet Sci. 2022 Dec 5;9(12):674. doi: 10.3390/vetsci9120674 (PMC9788024; doi:10.3390/vetsci9120674)

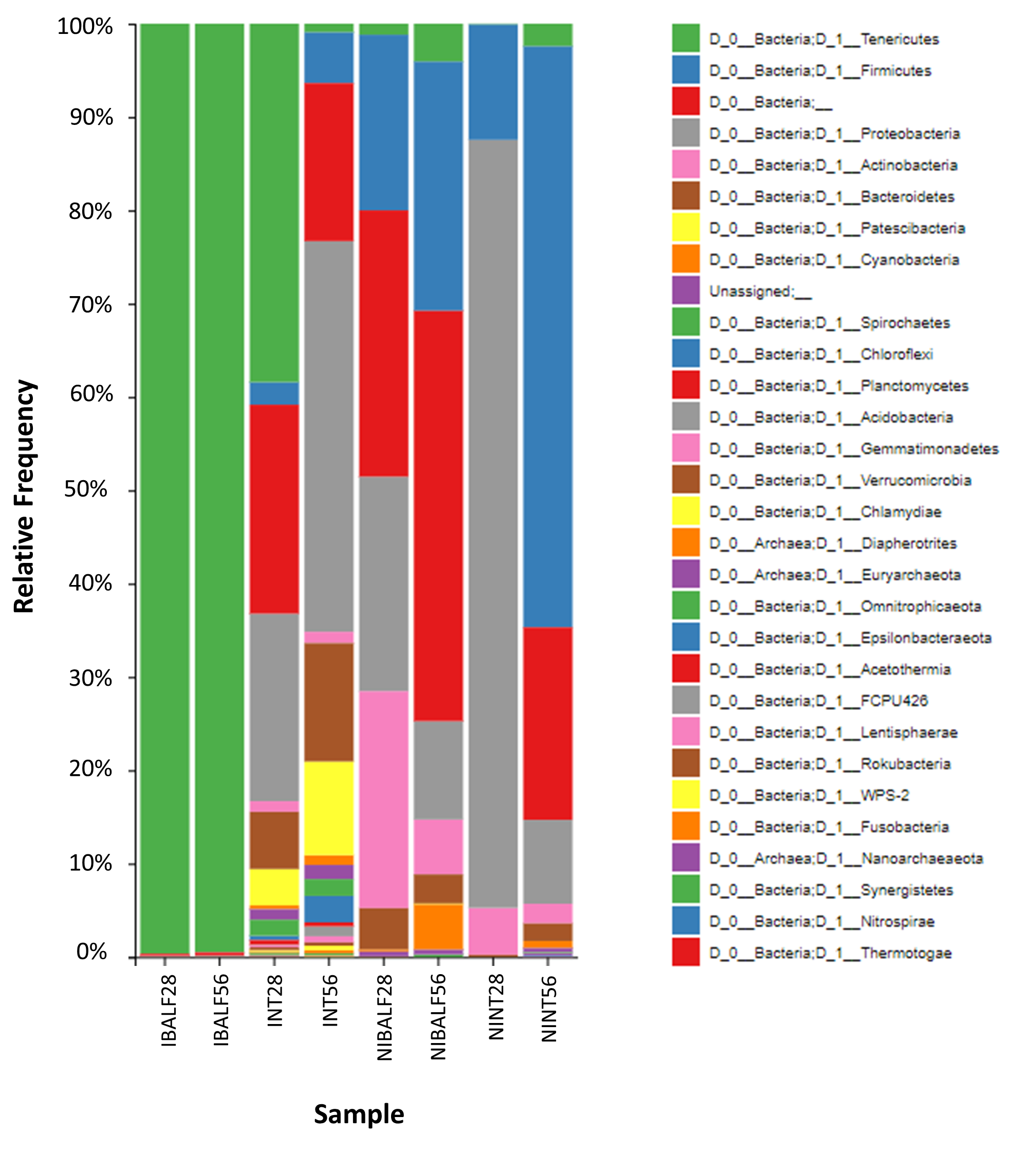

Supplement: Supplementary file 1 [file vetsci-09-00674-s001.zip › Figure S1.png]
